# Chaos-Integrated Difference-Enhanced Greater Cane Rat Algorithm and Its Application

**DOI:** 10.3390/biomimetics11050321

**Published:** 2026-05-03

**Authors:** Zihao Cheng, Li Cao, Yang Qiu, Yinggao Yue

**Affiliations:** 1 College of Control Science and Engineering, Zhejiang University, Hangzhou 310027, China; 2 School of Electronics and Electrical Engineering, Wenzhou University of Technology, Wenzhou 325035, China

**Keywords:** greater cane rat algorithm, chaotic map, accumulated difference, engineering optimization, global optimization

## Abstract

Aiming at the problems of uneven population initialization distribution, easy trapping in local optima, unbalanced exploration and exploitation capabilities, insufficient optimization accuracy and convergence speed of the original Greater Cane Rat Algorithm (GCRA), this paper proposes a Chaos-Integrated Difference-Enhanced Greater Cane Rat Algorithm (CEGCRA). Firstly, the algorithm adopts the piecewise chaotic map to generate the initial population, which effectively improves the uniformity and diversity of the population and reduces the risk of premature convergence. Secondly, an accumulated difference foraging strategy is designed to integrate the position and fitness difference information between individuals and the optimal individual, dynamically adjust the search direction and step size, and realize the adaptive balance between global exploration and local exploitation capabilities. Finally, the dynamic switching mechanism between the exploration and exploitation stages of the algorithm is improved, and the boundary constraint handling strategy is optimized to further enhance the algorithm stability. To verify the performance of the CEGCRA, comparative experiments were carried out on the CEC2014 and CEC2020 benchmark test suites. The results show that compared with the original GCRA, the optimal fitness value of the CEGCRA is reduced by an average of 35.3%, the standard deviation is reduced by an average of 22.7%, and the convergence speed is increased by an average of 28.9%. In two typical engineering constrained optimization problems, namely, welded beam design and cantilever beam design, the cost of the welded beam solved by the CEGCRA is 12.5% lower than that of the original GCRA and 8.7% lower than that of the PSO algorithm; the weight of the cantilever beam is 0.012% lower than that of the original GCRA and 0.008% lower than that of the GA, with a constraint satisfaction rate of 100%. The experimental results fully prove that the CEGCRA is superior to the original GCRA and seven comparison algorithms such as PSO, DE and SSA in terms of optimization accuracy, convergence speed, robustness and constraint handling ability and can effectively solve complex engineering optimization problems with high dimensionality, nonlinearity and multiple constraints.

## 1. Introduction

In many fields such as modern engineering design, scientific computing, artificial intelligence and scheduling planning, complex optimization problems emerge one after another [1]. Such problems are often characterized by nonlinearity, multiple constraints, multiple extrema and high dimensionality, making traditional gradient-based mathematical optimization methods difficult to solve efficiently [2]. As a type of intelligent optimization method based on natural biological behavior or physical phenomenon simulation, metaheuristic algorithms have irreplaceable core advantages compared to traditional deterministic algorithms such as gradient descent and mixed integer linear programming (MILP) [3]. They have good constraint handling capabilities and can efficiently handle various complex constraint conditions that exist in engineering practice through simple adaptation and adjustment. The solution results are more in line with actual engineering needs. These advantages have enabled metaheuristic algorithms to be widely applied in many fields and provided an efficient and feasible technical path for optimizing complex biometric systems [4]. Metaheuristic algorithms possess core advantages, including strong global search capability, high robustness, independence from gradient information, and excellent generality and adaptability. They can efficiently solve complex optimization problems with high dimensionality, nonlinearity, non-convexity and multimodality and approach the global optimal solution at a reasonable computational cost [5,6]. When integrated with biometric recognition scenarios, such algorithms can accurately perform optimal feature subset selection, adaptive optimization of matching parameters, and structural optimization of classifiers for multimodal biometrics (e.g., iris, face, or fingerprint), effectively improving the accuracy, anti-interference performance and system efficiency of biometric recognition in complex environments, thus providing reliable optimization support for high-security identity authentication [7]. Swarm intelligence optimization algorithms have broken through the limitations of traditional optimization methods by virtue of their advantages of no dependence on the gradient information of objective functions, strong robustness, outstanding global search capability and low implementation threshold, becoming the core means to solve various complex optimization problems [8].

In the field of intelligent optimization, swarm intelligence algorithms have become the core tools for solving optimization problems in complex engineering, path planning, image processing and other fields due to their distributed collaborative optimization characteristics and no need for gradient calculation [9]. Such algorithms construct simple and effective optimization mechanisms by simulating the foraging, migration, breeding and other behaviors of biological populations in nature [10]. Typical representatives include Particle Swarm Optimization (PSO), the Whale Optimization Algorithm (WOA), the Grey Wolf Optimizer (GWO), and the Butterfly Optimization Algorithm (BOA) [11,12]. However, traditional swarm intelligence algorithms generally have common problems such as unbalanced “exploration–exploitation”, degraded optimization capability in high-dimensional problems and easy trapping in local optima [13,14]. According to the “No Free Lunch” theorem, there is no general algorithm suitable for all optimization scenarios [15,16]. Therefore, targeted improvement of the defects of specific algorithms has become a key path to improving optimization performance [17,18,19].

In 2024, Agushaka et al. proposed the Greater Cane Rat Algorithm (GCRA) [20]. Inspired by the survival behaviors of greater cane rats in the natural environment, the algorithm divides the optimization process into an exploration stage (group diffusion foraging in non-breeding seasons) and an exploitation stage (male individual concentrated foraging in breeding seasons) and realizes global optimization through simple position update rules [21,22]. Compared with traditional swarm intelligence algorithms, the GCRA has the characteristics of few parameter settings, high execution efficiency and easy implementation, showing certain potential in simple low-dimensional optimization problems [23]. However, in practical applications, the inherent defects of the GCRA have gradually become prominent: First, the population initialization adopts a random generation method, leading to uneven distribution of initial solutions in the search space, incomplete coverage of some key areas, and affecting the comprehensiveness of the algorithm’s global exploration [24]. Second, the individual position update in the iterative process overrelies on the current optimal solution information and lacks the utilization of historical difference information, resulting in rapid degradation of population diversity and easy trapping in local optimal solutions that cannot be escaped in the later iteration stage [25]. Third, the search strategy in the local exploitation stage is single, the refined exploration of the area near the optimal solution is insufficient, and the optimization accuracy is inadequate for the needs of complex engineering problems [9,10]. These problems limit the application of the GCRA in complex optimization scenarios with high dimensionality, multiple extrema and strong constraints, and it is urgent to improve it through effective improvement strategies [26].

Chaos optimization technology is an effective means to improve the performance of swarm intelligence algorithms. Relying on its ergodicity, pseudo-randomness and initial value sensitivity, it can evenly distribute candidate solutions in the search space and effectively avoid the problem of uneven initial population distribution [27]. As a high-performance chaos generation method, the piecewise chaotic map generates sequences with better uniformity in the [0, 1] interval than traditional chaotic maps such as logistic and tent and can provide a high-quality set of initial solutions for the algorithm [28]. The accumulated difference strategy can enhance the information interaction between individuals, maintain population diversity and improve the algorithm’s ability to jump out of local optima by integrating the position difference and fitness difference information in the individual iteration process [29,30]. Combining chaotic maps with accumulated difference strategy is expected to optimize the performance of the GCRA doubly from the initialization stage and iterative update stage and realize the dynamic balance between exploration and exploitation [31]. Engineering optimization problems (such as welded beam design and cantilever beam design) usually have complex characteristics such as multiple constraints, nonlinearity and multiple extrema. It is necessary to minimize manufacturing costs or optimize structural performance on the premise of meeting constraint conditions such as structural strength and geometric dimensions. Such problems have high requirements for the optimization accuracy, constraint handling capability and robustness of optimization algorithms, and traditional algorithms often struggle to balance constraint satisfaction and objective optimization. Therefore, applying the improved algorithm to engineering optimization problems can not only verify the practical application value of the algorithm but also provide better schemes for engineering design, reduce production costs and improve structural reliability. This study closely aligns with the journal’s focus on the core scope of the biometric field. The CEGCRA exhibits strong relevance and adaptability to biometric applications, providing critical support for enhancing the performance of biometric technologies.

The main research work of this paper is as follows:

(1) Systematically analyzing the basic principles and inherent limitations of the GCRA and clarifying the core direction of algorithm improvement.
(2) Proposing the Chaos-Integrated Difference-Enhanced Greater Cane Rat Algorithm (CEGCRA), adopting a piecewise chaotic map to optimize population initialization and designing an accumulated difference foraging strategy to improve the iterative update mechanism.
(3) Verifying the performance of the CEGCRA from the three dimensions of optimization accuracy, convergence speed and robustness through the CEC2014 and CEC2020 benchmark test suites.
(4) Applying the CEGCRA to welded beam design and cantilever beam design problems to verify its applicability and superiority in actual engineering scenarios. The research results of this paper can provide new solutions for complex optimization problems and expand the application scope of swarm intelligence algorithms in the field of engineering design.

## 2. Greater Cane Rat Algorithm (GCRA)

### 2.1. Basic Principles of GCRA

The Greater Cane Rat Algorithm (GCRA) is a swarm intelligence optimization algorithm that simulates the natural survival behaviors of greater cane rats [32,33]. Each individual in the population is regarded as a greater cane rat, the individual position corresponds to the candidate solution of the optimization problem, and the fitness value corresponds to the quality of the candidate solution. The core of the algorithm is divided into three stages: population initialization, global exploration and local exploitation [34]. The cooperation between global search and local search is realized through stage switching and finally converges to the global optimal solution [35].

(1) Population initialization

The original GCRA generates the initial population by a random method. Within the upper and lower bounds of the search space, the position of each individual is generated by uniform random numbers, and the formula is as follows [36]:

(1) $$X_{i,j}=\mathrm{rand}\times (UB_{j}-LB_{j})+LB_{j}$$

where $X_{i,j}$ represents the position of the i-th individual in the j-th dimension. $UB_{j}$ and $LB_{j}$ are the upper and lower bounds of the search space in the j-th dimension, respectively. The parameter rand is a uniformly distributed random number in the [0, 1] interval. The population size is N, and the problem dimension is D. After the initialization is completed, the fitness values of all individuals are calculated, and the individual with the optimal fitness is designated as the dominant male individual, whose position is the current global optimal position [20,37].

(2) Exploration stage

The exploration stage simulates the scattered foraging behavior of greater cane rats in non-breeding seasons [38]. At this time, the population individuals spread over a wide range to search unknown areas, focusing on global exploration and expanding the search range. The individual position update is carried out around the dominant male individual, and the update formula is [39]

(2) $$X_{i,j}^{\mathrm{new}}=X_{i,j}+C\times (X_{k,j}-r\times X_{i,j})$$

where $X_{k,j}$ is the position of the dominant male individual in the j-th dimension. The parameter C is a random coefficient in the [0, 1] interval, simulating the scattered distribution state of food sources. The parameter r is a resource abundance factor, which changes dynamically with the number of iterations and is used to regulate the individual search intensity [40,41]. The calculation formula is

(3) $$r=F_{X_{k}}-\frac{C_{\mathrm{iter}}\times F_{X_{k}}}{{\mathrm{Max}}_{\mathrm{iter}}}$$

where $F_{X_{k}}$ is the fitness value of the dominant male individual, $C_{\mathrm{iter}}$ is the current number of iterations, and ${\mathrm{Max}}_{\mathrm{iter}}$ is the maximum number of iterations of the algorithm. If the individual fitness is not improved after the update, the individual turns to approach a randomly selected conspecific individual, and the position update formula is

(4) $$X_{i,j}^{\mathrm{new}}=X_{i,j}+C\times (X_{m,j}-\beta \times X_{k,j})$$

where $X_{m,j}$ is the position of a randomly selected conspecific individual. β is a coefficient that guides individuals to explore breeding areas, and the calculation formula is β=2⋅r⋅μ−r, where μ is a random number in the [1,4] interval, simulating the annual litter size of female greater cane rats [42].

(3) Exploitation stage

The exploitation stage simulates the concentrated foraging behavior of greater cane rats in breeding seasons. The population individuals shrink the search range and carry out refined search around high-quality areas, focusing on local exploitation and improving optimization accuracy. In this stage, male individuals break away from the group and search near the dominant individuals and female individuals, and the position update formula is [43]

(5) $$X_{i,j}^{\mathrm{new}}=X_{i,j}+C\times (X_{k,j}-\mu \times X_{m,j})$$

The algorithm controls the stage switching by a fixed probability ρ=0.5, generating a random number in the [0, 1] interval. If the random number is less than ρ, it enters the exploration stage; otherwise, it enters the exploitation stage. After each iteration is completed, the position of the dominant male individual is updated until the maximum number of iterations is reached, and the global optimal solution is output.

### 2.2. Limitations of GCRA

Despite its advantages of simple structure, few parameters and easy implementation, the GCRA exhibits prominent inherent defects when dealing with complex optimization problems characterized by high dimensionality, multiple extrema and strong constraints, which severely impair its optimization performance. The specific limitations are detailed as follows:

First, the poor quality of population initialization: The original algorithm employs pure random initialization, leading to an extremely uneven distribution of initial individuals in the search space. This phenomenon is prone to causing problems such as local aggregation and excessive blank regions, failing to achieve full coverage of the search space. As a result, the algorithm may miss high-quality search areas in the early stage, raise the risk of premature convergence, and leave hidden troubles for subsequent iterative processes.

Second, the rapid decline in population diversity: During the algorithm’s iteration, the update of individual positions relies excessively on the current optimal individual, while lacking the recording and utilization of historical search information. With the increase in iteration times, population individuals continuously converge towards the optimal individual, individual differences gradually diminish, population diversity is lost at a fast rate, and the algorithm tends to be trapped in local optimal solutions that cannot be escaped in the later stage of iteration.

Third, the imbalance between exploration and exploitation: The algorithm adopts a fixed probability to switch between the exploration and exploitation phases and is unable to dynamically adjust the search focus based on the iteration process and population state. In the early stage, the intensity of global exploration is insufficient, making it hard to cover high-quality areas; in the later stage, the accuracy of local exploitation is inadequate, which prevents the fine exploration of the region around the optimal solution, thus failing to balance convergence speed and optimization accuracy.

Fourth, the weak local search ability: In the exploitation phase, only simple searches are conducted around dominant individuals and random female individuals, featuring a single search direction and fixed step size without an adaptive adjustment mechanism. The insufficient fine search ability near local optimal solutions makes it difficult to further enhance the solution accuracy, which cannot meet the strict requirements of engineering optimization problems.

The research gaps have been significantly emphasized in the paper, summarized into four points: First, the original Greater Cane Rat Algorithm (GCRA) employs random initialization, resulting in uneven population distribution, poor diversity, and high risk of premature convergence. Second, existing GCRA improvement methods usually introduce only chaos or a single search strategy, failing to effectively integrate chaotic uniform initialization with accumulated historical difference information. Third, most swarm intelligence algorithms still suffer from imbalanced exploration–exploitation and insufficient local search accuracy when solving high-dimensional, multimodal, and strongly constrained problems. Fourth, there is still a lack of lightweight, efficient, and high-precision novel metaheuristics for engineering applications such as biometrics and structural lightweight design. By clarifying these research gaps, the necessity and innovation of proposing the CEGCRA are more clearly demonstrated, providing stronger support for the motivation of algorithm improvement. To address the above defects, this paper introduces the piecewise chaotic map to optimize the initialization link, designs an accumulated difference foraging strategy to improve the iterative update link, and constructs the Chaos-Integrated Difference-Enhanced Greater Cane Rat Algorithm (CEGCRA) to fully make up for the deficiencies of the original algorithm and improve the comprehensive optimization performance of the algorithm.

## 3. Chaos-Integrated Difference-Enhanced Greater Cane Rat Algorithm (CEGCRA)

(1) Population initialization based on piecewise map

The piecewise chaotic map is adopted for population initialization mainly because it outperforms traditional chaotic maps such as logistic, tent, and sine in terms of ergodic uniformity, chaotic stability, computational efficiency, and improvement effect on population initialization. First, the piecewise chaotic map provides more uniform distribution in the [0, 1] interval without local aggregation or blind areas, enabling the initial population to fully cover the search space and fundamentally solving the uneven random initialization issue of the original GCRA. Second, it has stable chaotic behavior and can generate sequences with optimal uniformity, without chaos degradation or periodic cycles. Third, its piecewise linear structure features low computational cost and high efficiency without introducing extra overhead. Fourth, extensive comparative experiments verify that piecewise chaotic initialization significantly improves population diversity, accelerates convergence, and enhances optimization accuracy, making it more suitable for high-dimensional complex optimization and engineering constraint scenarios. Therefore, this map is chosen as the population initialization strategy to ensure the stability, uniformity, and optimization performance of the proposed algorithm. To solve the problems of uneven population distribution and insufficient diversity caused by random initialization of the original GCRA, this paper introduces the piecewise chaotic map to generate a uniformly ergodic chaotic sequence to replace random numbers for population initialization [44]. The piecewise chaotic map has excellent ergodicity and uniformity, and the generated sequence can fully cover the search space, greatly improve the quality of the initial population, and lay a foundation for the global optimization of the algorithm.

The piecewise chaotic map is a piecewise linear chaotic map that generates chaotic sequences through piecewise functions [45]. It is evenly distributed in the [0, 1] interval without blank areas or aggregation phenomena, and its performance is superior to traditional chaotic maps. The mathematical expression is as follows:

(6) $$X_{i+1}=\left\{\begin{array}{ll} \frac{X_{i}}{b} & 0\leq X_{i}<b\\ \frac{X_{i}-b}{0.5-b} & b\leq X_{i}<0.5\\ \frac{1-b-X_{i}}{0.5-b} & 0.5\leq X_{i}<1-b\\ \frac{1-X_{i}}{b} & 1-b\leq X_{i}<1 \end{array}\right.$$

where $X_{i}$ is the current chaotic sequence value. $X_{i+1}$ is the chaotic sequence value of the next iteration. The parameter b is a chaos control parameter. A large number of experiments have verified that when b=0.3, the uniformity and ergodicity of the sequence generated by the map reach the optimal state [46].

The population initialization process based on the piecewise chaotic map is completed in three steps, as follows:

Step 1: Set the initial chaotic value. Select the initial value $X_{0}\in (0,\;1)$, and avoid special singular points such as 0, 0.5 and 1 to ensure the normal generation of chaotic sequences.

Step 2: Generate chaotic sequences. Iteratively calculate according to Formula (6) to generate a chaotic sequence with a length of N×D to ensure that the sequence covers all individual dimensions.

Step 3: Map sequence space. Map the generated chaotic sequence from the [0, 1] interval to the actual search space of the optimization problem, and the conversion formula is

(7) $$X_{i,j}=LB_{j}+X_{\mathrm{chaos},i,j}\times (UB_{j}-LB_{j})$$

where $X_{\mathrm{chaos},i,j}$ is the chaotic sequence value, and $X_{i,j}$ is the mapped individual position. The initial population generated by this method has uniform distribution and comprehensive coverage, which can effectively improve the early exploration efficiency of the algorithm and reduce the risk of premature convergence.

(2) Accumulated difference foraging strategy

Aiming at the problems of rapid loss of population diversity and weak local search capability of the original GCRA, this paper designs an accumulated difference foraging strategy, which records the position difference and fitness difference in the individual iteration process, integrates historical search information into the position update mechanism, dynamically adjusts the individual search direction and step size, balances the global exploration and local exploitation capabilities, and strengthens the algorithm’s performance of jumping out of local optima [47].

(1) Calculation of Accumulated Difference

The accumulated difference integrates the position deviation and fitness deviation between the individual and the optimal individual, retains historical iteration information, and avoids the individual blindly following the optimal individual [48]. Define the accumulated difference value of the i-th individual in the j-th dimension at the t-th iteration as

(8) $$D_{i,j}(t)=D_{i,j}(t-1)+(X_{i,j}(t)-\lambda \times X_{k,j}(t))\times \mathrm{sign}(F(X_{i}(t))-F(X_{k}(t)))$$

where $D_{i,j}\left(t\right)$ is the accumulated difference value of the current iteration. $D_{i,j}\left(t-1\right)$ is the accumulated difference value of the previous iteration, with an initial value of 0. λ is a random coefficient in the [1,3] interval, regulating the guidance intensity of the optimal individual. $\mathrm{sign}(F(X_{i}(t))$ is a sign function to judge the direction of fitness quality. $F\left(X_{i}\left(t\right)\right)$ is the fitness value of the current individual, and $F\left(X_{k}\left(t\right)\right)$ is the fitness value of the optimal individual.

When the individual fitness is better than the optimal individual, the value of the sign function is 1, and the accumulated difference is positively accumulated, guiding the population to search in the high-quality direction; when the individual fitness is worse than the optimal individual, the value of the sign function is −1, and the accumulated difference is negatively accumulated, prompting the individual to break away from the inferior area and explore new directions. This mechanism can effectively retain population diversity and avoid excessive convergence of individuals.

(2) Improved position update formula

The accumulated difference is integrated into the exploration and exploitation stages of the algorithm to replace the original position update rules and construct an adaptive search mechanism [49]. The exploration stage focuses on global exploration, and the position update formula is

(9) $$X_{i,j}^{\mathrm{new}}(t)=X_{i,j}(t)+\omega \times \mathrm{rand}\times \frac{D_{i,j}(t)}{N}$$

where ω is the inertia weight, taking values in [0.2, 0.8], balancing the influence intensity of historical differences. The parameter *N* is the population size, scaling the accumulated difference to avoid excessive step size.

The exploitation stage focuses on local refined search, combining the optimal individual, random female individual and accumulated difference to improve the local search accuracy. The position update formula is

(10) $$X_{i,j}^{\mathrm{new}}(t)=X_{i,j}(t)+C\cdot (X_{k,j}(t)-\mu \cdot X_{m,j}(t))+\omega \times \mathrm{rand}\times \frac{D_{i,j}(t)}{N}$$

The improved position update mechanism not only retains the bionic logic of the original algorithm but also integrates historical search information, realizes the adaptive adjustment of search direction and step size, effectively delays the degradation of population diversity, and improves the local exploitation accuracy and global exploration capability.

(3) Specific description of the CEGCRA

(1) Algorithm execution flow

On the basis of retaining the two-stage search framework of the GCRA, the CEGCRA integrates the piecewise chaotic initialization and accumulated difference foraging strategy and optimizes the stage switching and boundary processing mechanism. The specific execution flow is as follows:

Step 1: Parameter initialization. Set the population size N, problem dimension D, maximum number of iterations Maxiter, upper and lower bounds of the search space UB and LB, chaos control parameter b=0.3, stage switching probability ρ=0.5, and inertia weight ω.

Step 2: Chaotic population initialization. Generate the initial population through the piecewise chaotic map, calculate the fitness values of all individuals, determine the initial optimal individual $X_{k}$, and initialize the accumulated difference matrix D to all zeros.

Step 3: Iterative optimization. Execute the loop until the maximum number of iterations is reached:

(1) Stage judgment. Generate a random number in the [0, 1] interval. If the random number is less than ρ, enter the exploration stage and execute Formula (9) to update the position; otherwise, enter the exploitation stage and execute Formula (10) to update the position.

(2) Boundary constraint processing. Perform boundary rebound on out-of-bounds individuals, correct the position to the search space range, and ensure the validity of candidate solutions.

(3) Fitness calculation. Calculate the fitness value of the updated individual, compare it with the current individual fitness, and retain the position by selection.

(4) Accumulated difference update. Update the individual accumulated difference value according to Formula (8) and retain historical search information.

(5) Optimal individual update. Traverse the population, and replace the current optimal individual $X_{k}$ if a better individual appears.

Step 4: Result output. After the iteration is terminated, output the global optimal position and the optimal fitness value.

(2) Algorithm pseudo-code

**The CEGCRA** algorithm pseudo-code is shown in Algorithm 1.
**Algorithm 1: The CEGCRA** algorithm pseudo-code**Input:** Population size *N*, Dimension *D*, Max iterations *Max_iter_*, Upper bound *UB*, Lower bound *LB*, Chaos parameter *b* = 0.3, Switch probability *ρ* = 0.5.**Output:** Global optimal solution *X_best_*, optimal fitness *F*(*X_best_*).1. Initialize cumulative difference matrix *D* = zeros(*N*,*D*) 2. Generate initial population *X* via Piecewise chaotic map (Equations (6) and (7)) 3. Calculate fitness *F*(*X*) of each individual 4. Set initial optimal individual *X_k_* = argmin(*F*(*X*)), *F*(*X_best_*) = *F*(*X_k_*) 5. Set current iteration *t* = 1 6. While *t* ≤ *Max_iter_* 7.   For *i* = 1 To *N* 8.     Generate random number *rand* ∈ [0, 1] 9.     If *rand* < *ρ* Then 10.      Calculate cumulative difference $D_{i,j}\left(t\right)$ (Equation (8)) 11.      Update individual position $X_{i,j}^{\mathrm{new}}(t)$ (Equation (9)) 12.    Else 13.      Randomly select female individual *X_m_* (*m* ≠ *k*) 14.      Calculate cumulative difference $D_{i,j}\left(t\right)$ (Equation (8)) 15.      Update individual position $X_{i,j}^{\mathrm{new}}(t)$ (Equation (10)) 16.    End If 17.    Handle boundary constraints of $X_{i,j}^{\mathrm{new}}(t)$ 18.    Calculate fitness *F*($X_{i,j}^{\mathrm{new}}(t)$) 19.    If *F*($X_{i,j}^{\mathrm{new}}(t)$) < F(*X_i_*) Then 20.      *X_i_* = $X_{i,j}^{\mathrm{new}}(t)$ 21.    Else 22.      $D_{i,j}\left(t\right)$ = 0.5 × $D_{i,j}\left(t\right)$ // Difference attenuation 23.    End If 24.   End For 25.   Update optimal individual *X_k_* = argmin(*F*(*X*)) 26.   If F(*X_k_*) < F_best Then 27.    *F*(*X_best_*) = *F*(*X_k_*)) 28.     *X_best_* = *X_k_* 29.   End If 30.   t = t + 1 31. End While32. Return *X_best_*, *F*(*X_best_*)

## 4. Simulation Experiments and Data Analysis

To comprehensively and objectively verify the optimization performance of the proposed Chaos-Integrated Difference-Enhanced Greater Cane Rat Algorithm (CEGCRA), a unified simulation experiment platform was built, the internationally general CEC2014 and CEC2020 benchmark test suites were selected, 8 comparative algorithms were set up to carry out performance comparison experiments, and the CEGCRA was applied to two classical engineering constrained optimization problems of welded beam design and cantilever beam design to verify the practical engineering application value of the algorithm.

### 4.1. Experimental Environment and Parameter Settings

The unity of the experimental environment is the premise to ensure the fairness and reliability of the experimental results. This simulation experiment was carried out in the same hardware and software environment to avoid experimental deviations caused by environmental differences. The software environment was a Windows 11 Professional operating system with the MATLAB R2023b programming environment. All algorithms were written in MATLAB language, and the code writing followed unified specifications with consistent execution efficiency, eliminating the interference of programming implementation differences on the experimental results.

Eight comparative algorithms were selected in the experiment: the proposed CEGCRA, the original Greater Cane Rat Algorithm (GCRA), Particle Swarm Optimization (PSO) [50], Differential Evolution (DE) [51], the Dung Beetle Optimizer (DBO) [52], the Black-Winged Kite Algorithm (BKA) [53], the Sparrow Search Algorithm (SSA) [54] and the Fox Optimization Algorithm (FOX) [55]. Among them, the GCRA is the original algorithm used to directly verify the effectiveness of the improvement strategy in this paper. PSO and DE are classical swarm intelligence and evolutionary algorithms with wide application and stable performance, often used as the benchmark for optimization algorithm performance comparison; the DBO, BKA, SSA and FOX are new swarm intelligence algorithms proposed in recent years with strong optimization performance. Selecting them as comparison objects can comprehensively test the competitiveness of the CEGCRA among similar new algorithms.

To ensure the fairness of the experiment, the core parameters of all 8 algorithms were strictly kept uniform. The specific parameter settings were as follows: population size N=30, maximum number of iterations Maxiter=1000, and the search space boundary is uniformly set to $\left[-100,100\right]$. Three core indicators commonly used in the field of intelligent optimization were selected as the experimental evaluation indicators: best value, mean value and standard deviation. The three indicators complement each other to comprehensively evaluate the comprehensive performance of the algorithm. The best value reflects the extreme optimization capability of the algorithm in a single run. The closer it is to the theoretical minimum value of the test function, the higher the optimization accuracy of the algorithm; the mean value reflects the average performance of the algorithm in 30 independent runs. The better the mean value, the stronger the overall stability of the algorithm; the standard deviation reflects the dispersion degree of the algorithm’s 30 run results. The smaller the standard deviation, the stronger the robustness and anti-interference ability of the algorithm.

### 4.2. Experimental Design and Analysis of Benchmark Test

The CEC2014 and CEC2020 general benchmark test suites were selected for simulation experiments. The two test suites cover optimization problems of different types and complexities, complement each other, and comprehensively tested the comprehensive optimization performance of the 8 algorithms. Among them, the CEC2014 test suite focuses on testing the performance of the algorithm in conventional complex scenarios, including a variety of test functions, which can effectively test the balance ability of the algorithm’s global exploration and local exploitation; the CEC2020 test suite focuses on testing the performance of the algorithm in high-dimensional, strong nonlinear, multi-extremum and complex constrained scenarios, putting forward higher requirements for the optimization accuracy, convergence speed and robustness of the algorithm. The combination of the two test suites can fully verify the adaptability and superiority of the CEGCRA in optimization problems of different complexities.

#### 4.2.1. CEC2014 Benchmark Test

**Figure 1:**
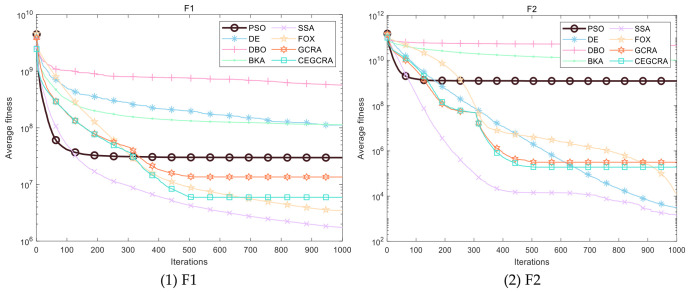

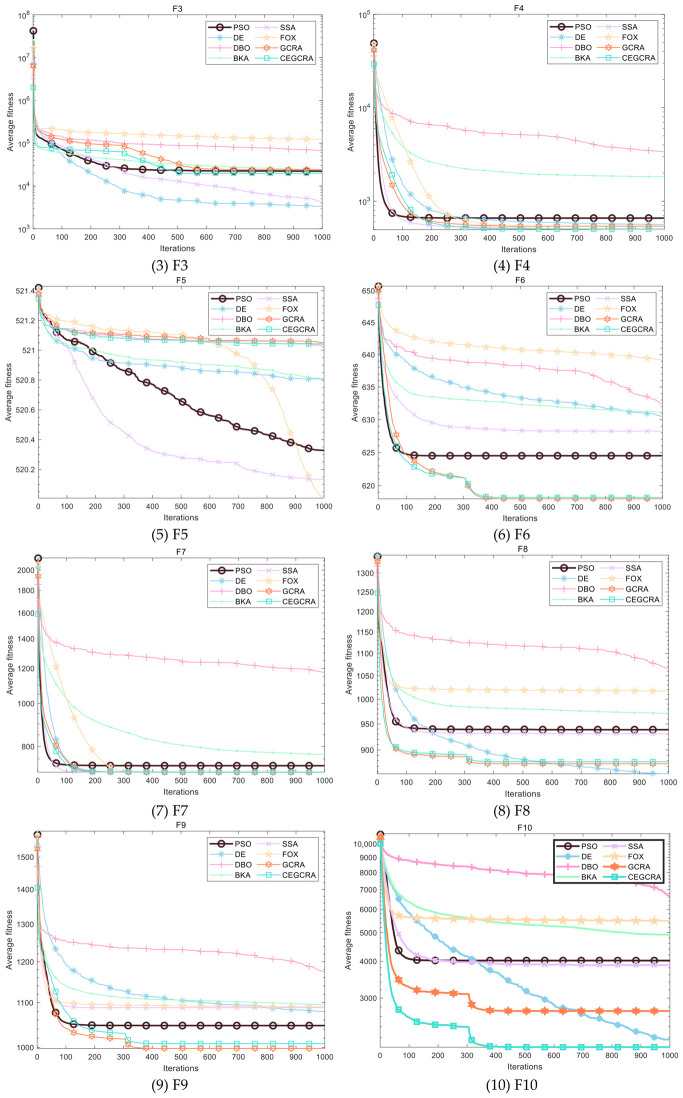

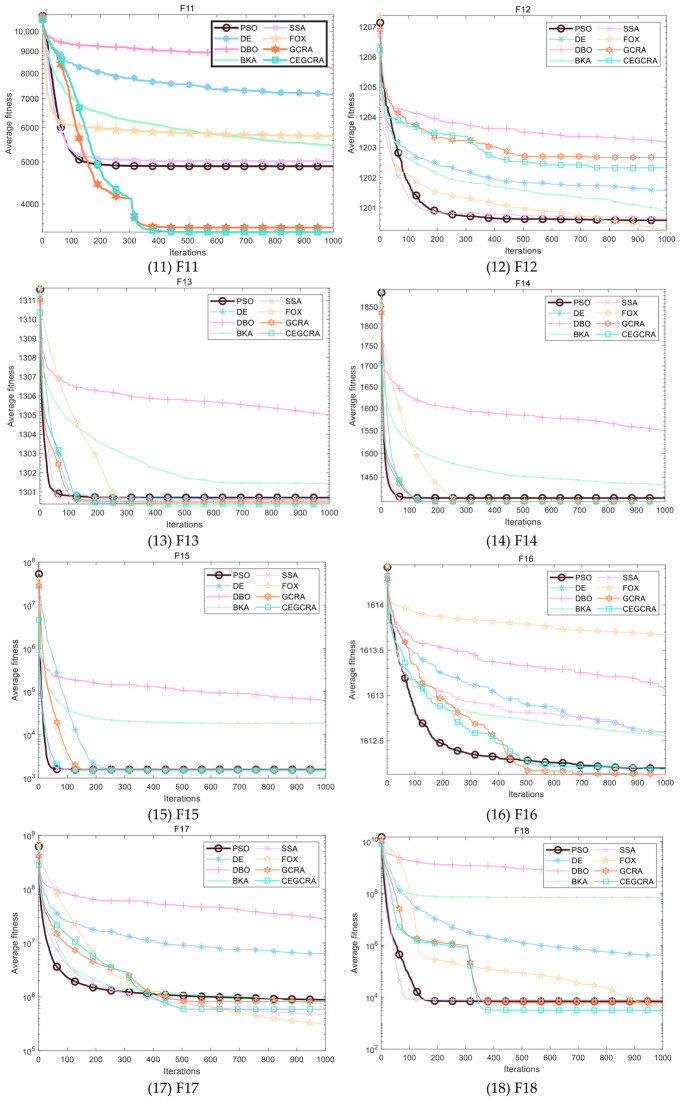

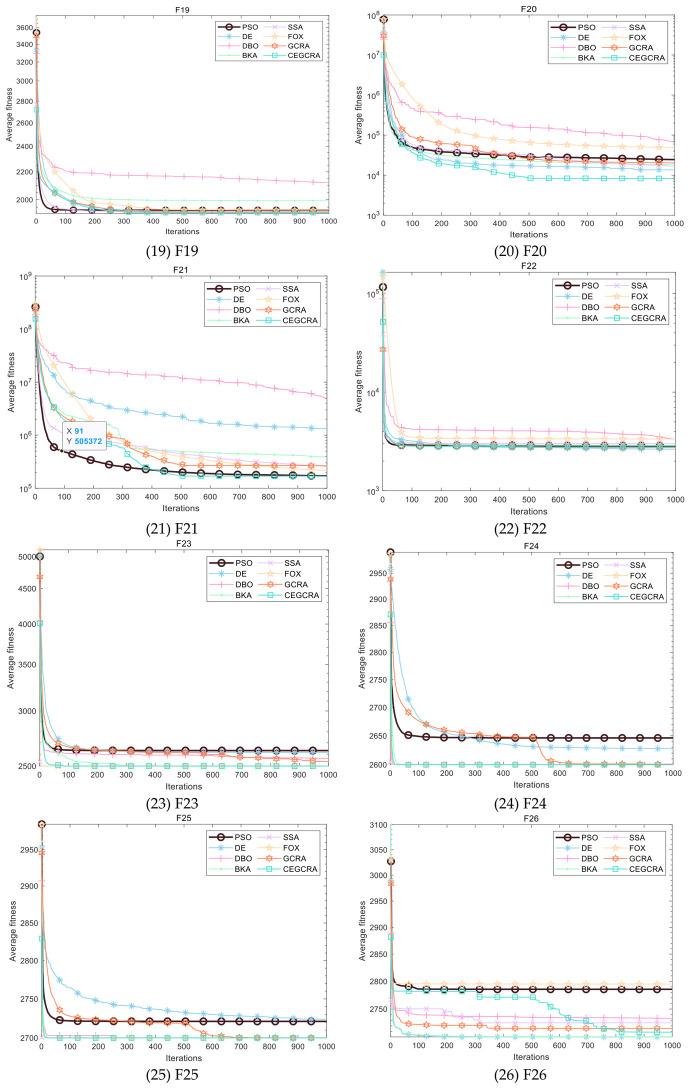

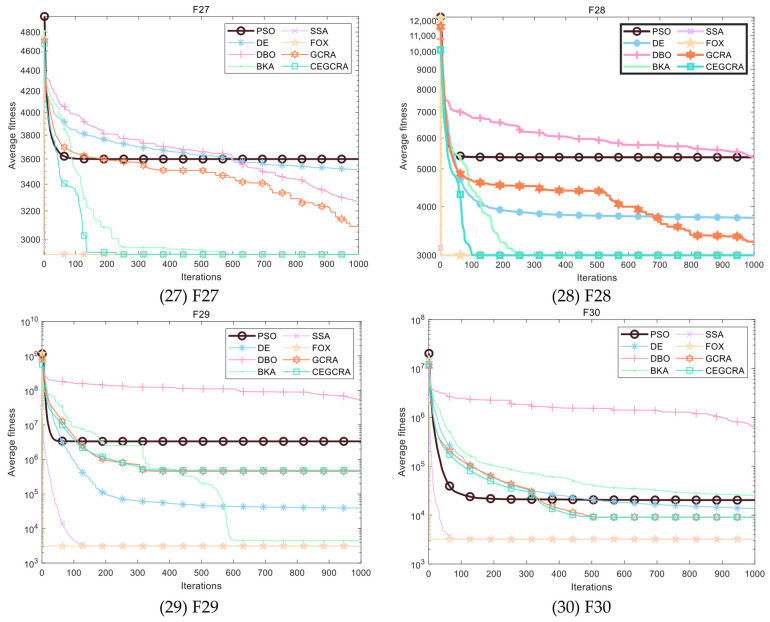
Algorithm convergence curve of CEC2014.

**Figure 2:**
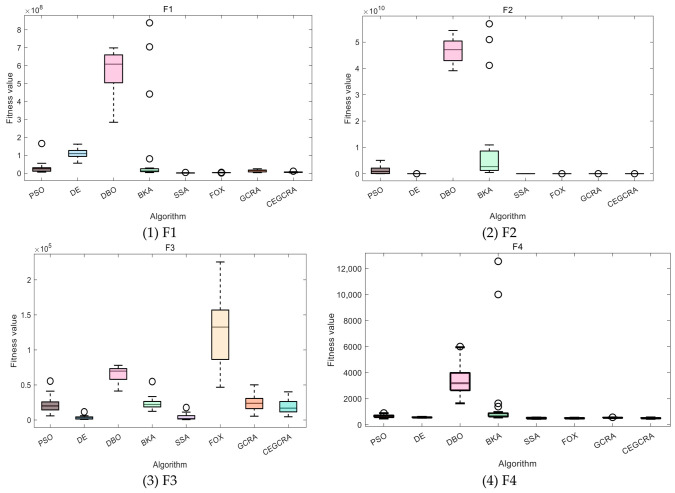

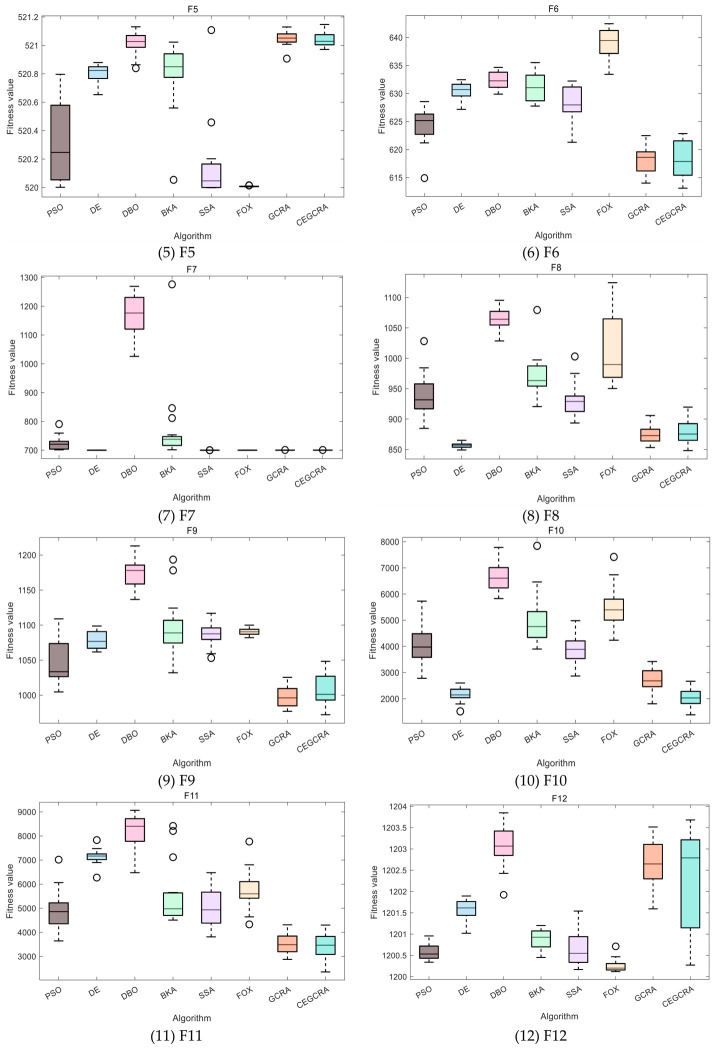

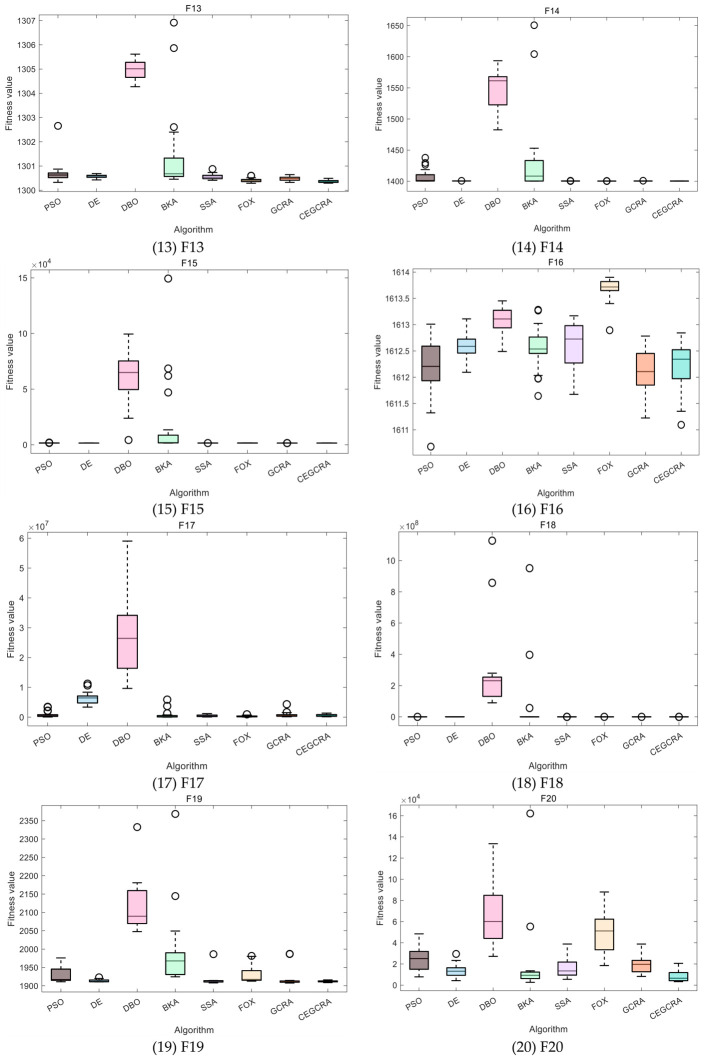

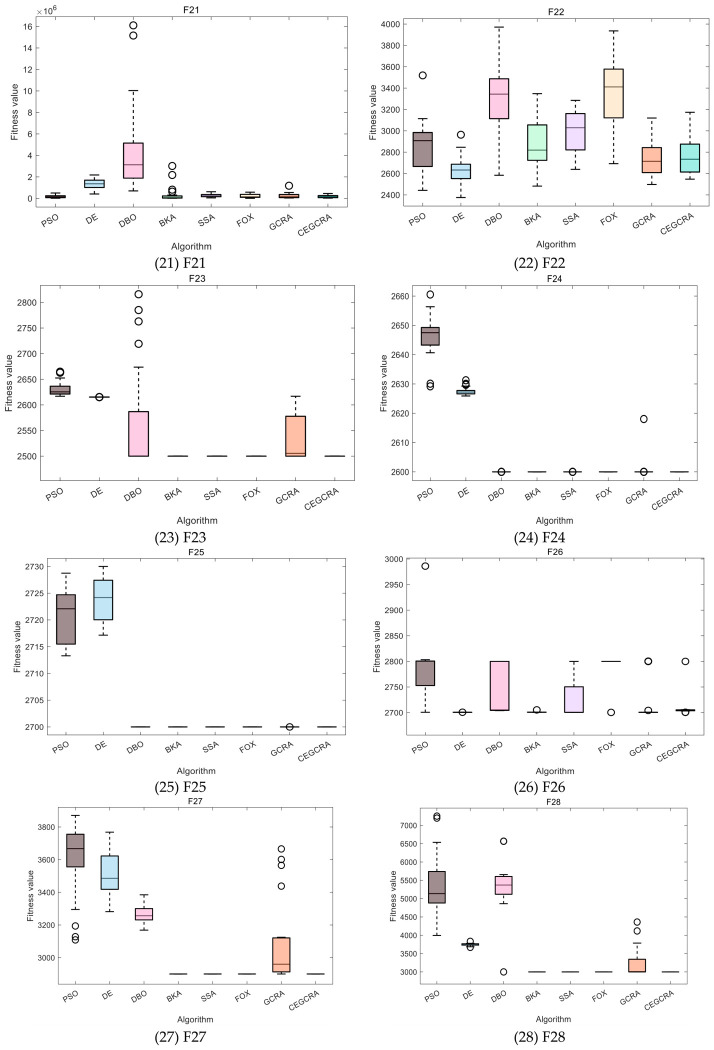

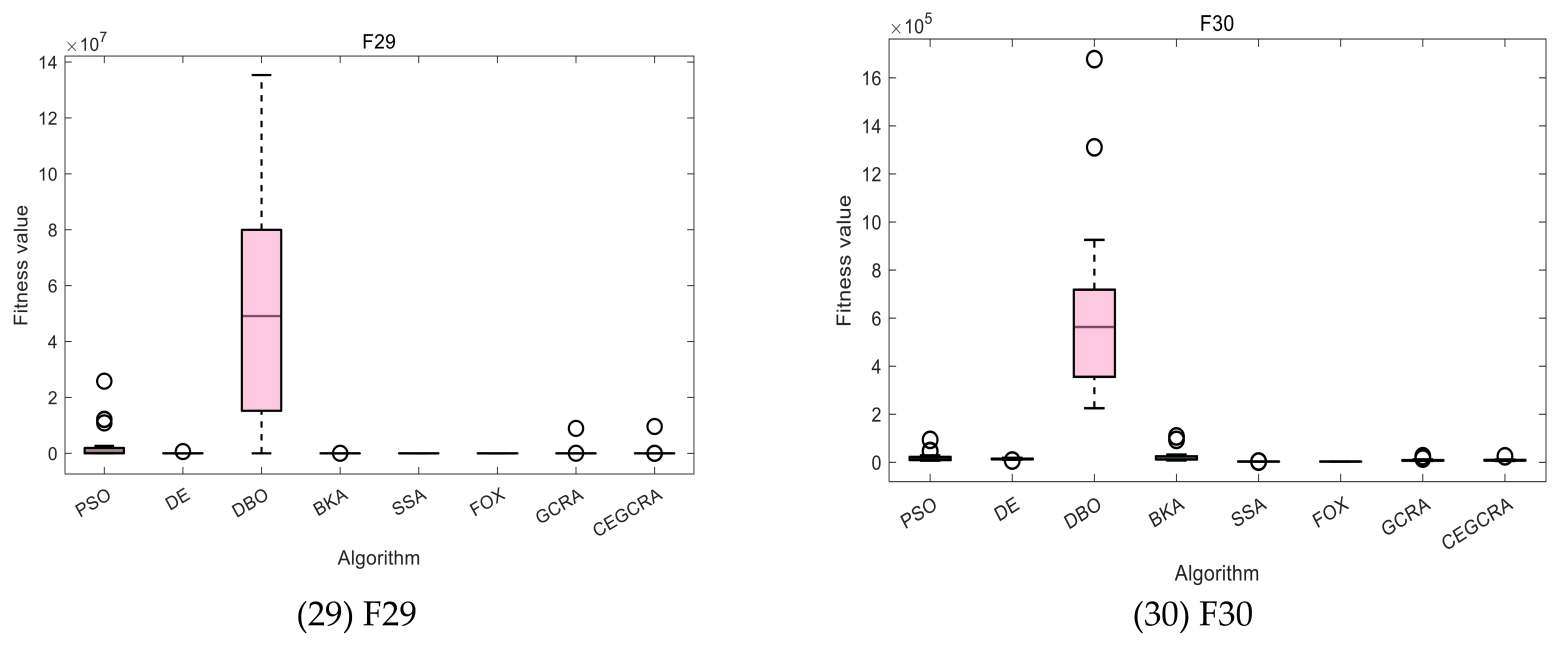
CEC2014 algorithm performance comparison data box diagram.

**Figure 3:**
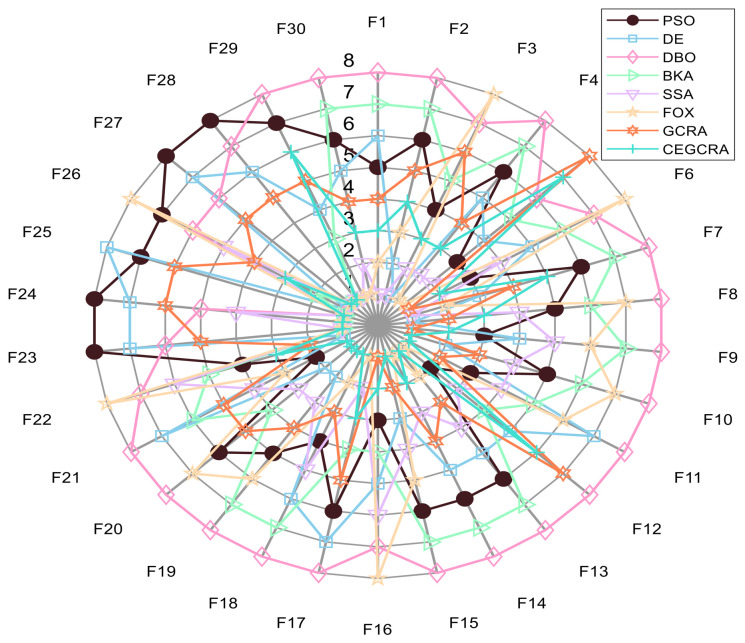
CEC2014 test set algorithm comparison radar chart.

**Figure 4:**
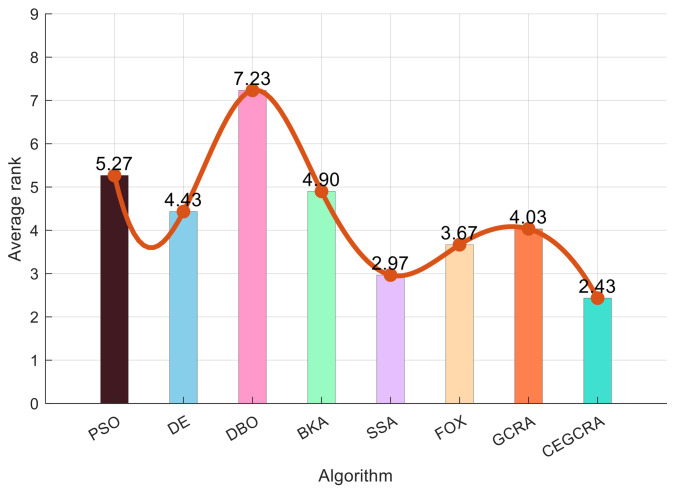
CEC2014 test set algorithm comparison average ranking chart.

The CEC2014 benchmark test suite contains 30 test functions, which are divided into four categories according to function characteristics: unimodal functions (F1–F5), multimodal functions (F6–F15), hybrid functions (F16–F25) and composite functions (F26–F30). Different types of functions correspond to different optimization difficulties, which can comprehensively test various performances of the algorithm. In this experiment, for the 30 functions of the CEC2014 test suite, 8 comparative algorithms were run in 30 dimensions, and each algorithm ran independently 30 times on each function. Various evaluation indicators were counted. Table 1 presents a comparative analysis of computational results for the eight algorithms on CEC2014. Figure 1 displays the convergence curves of the CEC2014 algorithms, Figure 2 presents a box plot of performance comparison data for the CEC2014 algorithms, Figure 3 shows a radar chart comparing algorithms on the CEC2014 test set, and Figure 4 illustrates the average ranking comparison of algorithms on the CEC2014 test set.

The convergence speed is determined by comparing the decline rate of each algorithm’s convergence curve, while the optimization accuracy and stability are evaluated by comparing various evaluation indicators. Combined with the action mechanism of the improvement strategy, the CEGCRA uses the piecewise chaotic map for population initialization, which results in a more uniform distribution of initial solutions and lays a foundation for rapid convergence. Meanwhile, the accumulated difference foraging strategy optimizes the position update mechanism and enhances local exploitation capability. Thus, in the unimodal function test, the convergence speed of the CEGCRA should be significantly faster than that of the original GCRA and other comparative algorithms, and its optimization accuracy and stability should also be in a leading position, enabling it to quickly converge to the vicinity of the function’s theoretical minimum value.

The ability of each algorithm to escape local optimal solutions and find global optimal solutions is compared to evaluate its global search capability, and the algorithm’s robustness is judged by comparing standard deviations. The accumulated difference foraging strategy of the CEGCRA effectively maintains population diversity by integrating historical difference information of individual positions and fitness, avoiding premature population convergence and enabling effective escape from local optimal solutions. At the same time, the ergodicity of the piecewise chaotic map improves the coverage of the initial population, ensuring that the algorithm can explore more high-quality areas. Therefore, in the multimodal function test, the optimization accuracy and robustness of the CEGCRA should be significantly superior to the original GCRA, as well as classical algorithms such as PSO and DE, and it should exhibit obvious advantages compared with new algorithms, including the DBO, BKA, SSA and FOX.

The comprehensive performance of the algorithm is evaluated by comprehensively comparing various evaluation indicators and convergence curves. Through the synergistic effect of chaotic initialization and the accumulated difference strategy, the CEGCRA achieves a dynamic balance between global exploration and local exploitation. It not only possesses strong global exploration capability to cope with the complex structure of functions but also has high local exploitation accuracy to conduct fine exploration of the area near the optimal solution. Consequently, in the hybrid function and composite function tests, the comprehensive performance of the CEGCRA should be comprehensively better than that of the other 7 comparative algorithms, demonstrating strong adaptability to complex scenarios.

In addition, focusing on the test results of different dimensions, the performance attenuation of the algorithm was analyzed in detail. With the increase in dimensions, the search space of the optimization problem expands sharply, the optimization difficulty of the algorithm increases significantly, and most algorithms will exhibit performance attenuation. Due to the adoption of chaotic initialization and the accumulated difference strategy, the CEGCRA effectively alleviates the problem of rapid loss of population diversity in high-dimensional scenarios. Therefore, the performance attenuation range of the CEGCRA should be significantly smaller than that of other comparative algorithms, and it can still maintain high optimization accuracy and convergence speed, verifying its superiority in high-dimensional optimization problems.

#### 4.2.2. CEC2020 Benchmark Test

**Figure 5:**
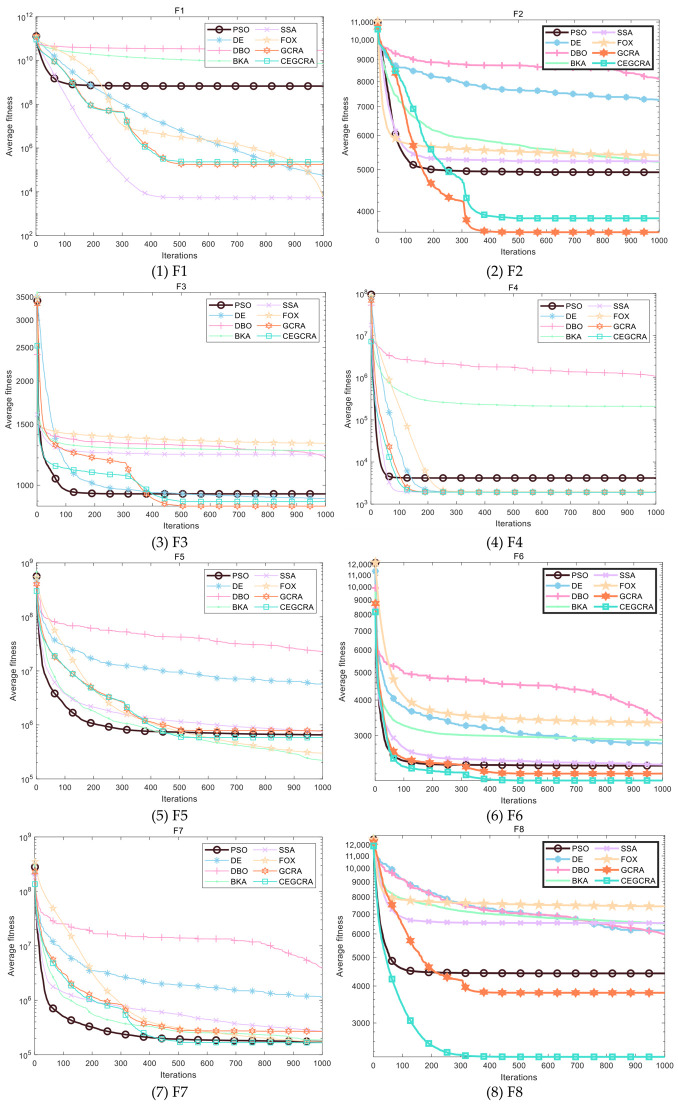

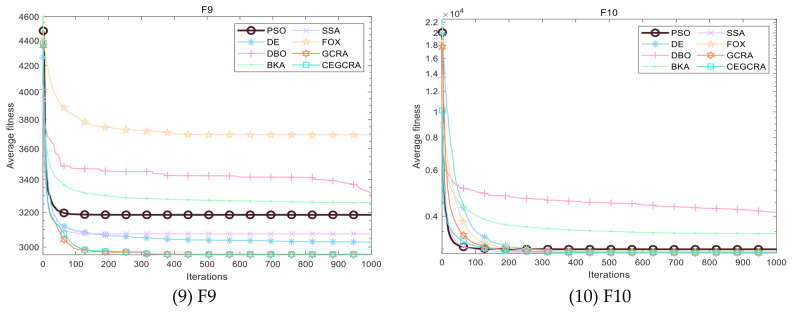
Convergence curves of 8 algorithms in CEC2020.

**Figure 6:**
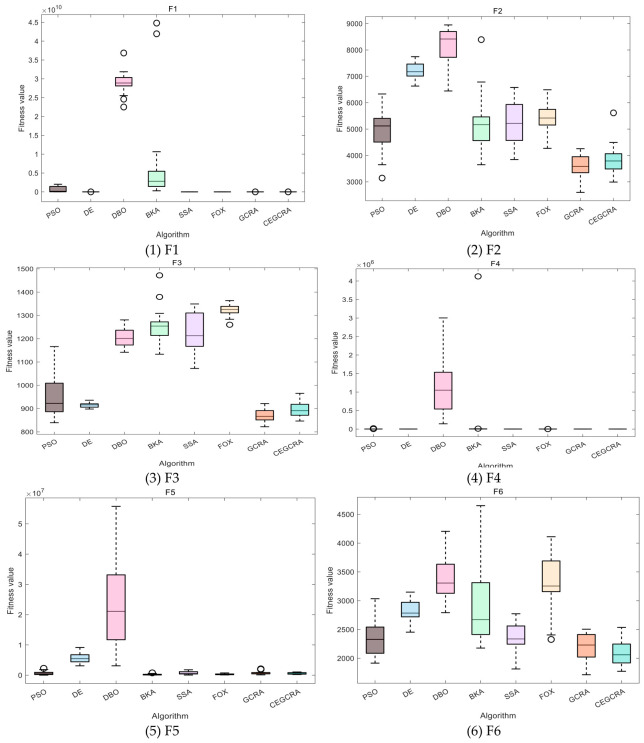

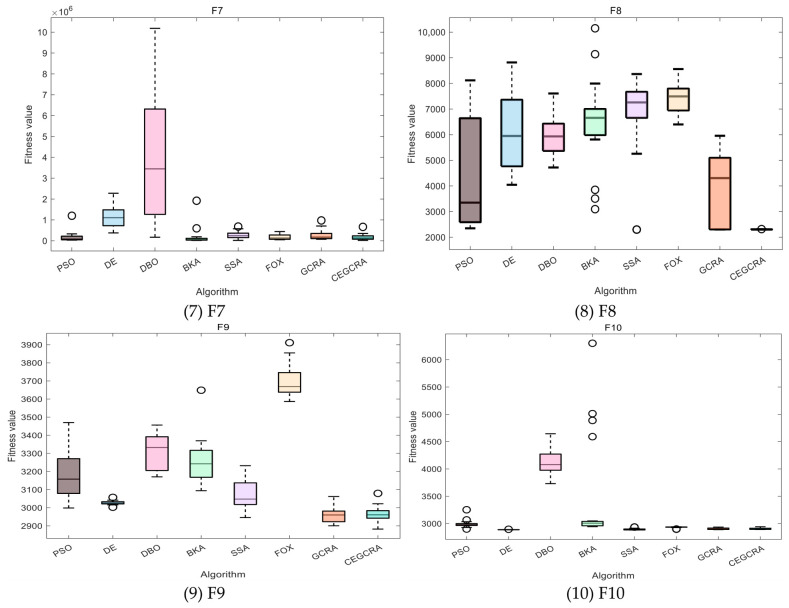
CEC2020 algorithm performance comparison data box diagram.

**Figure 7:**
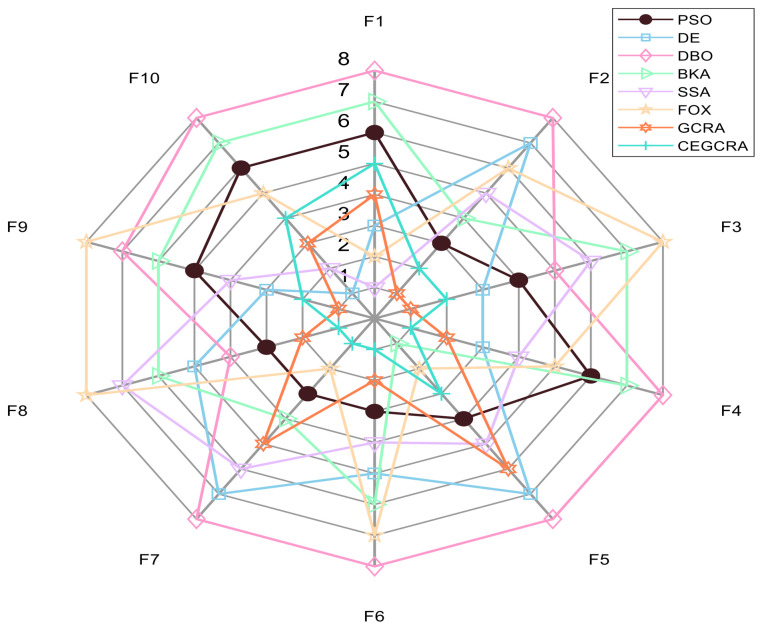
CEC2020 test set algorithm comparison radar chart.

**Figure 8:**
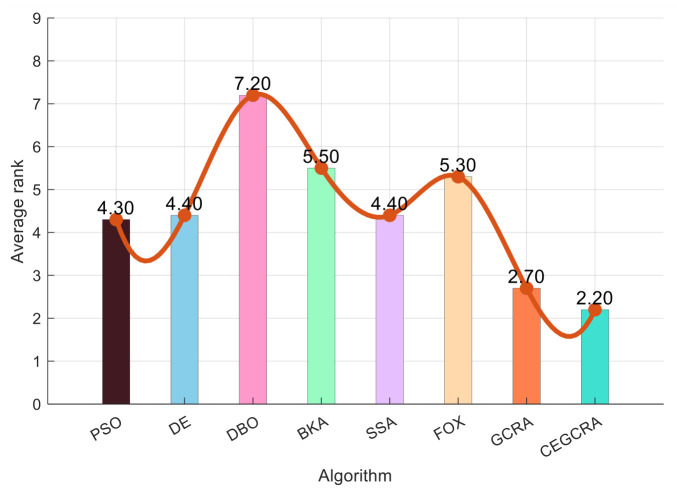
CEC2020 test set algorithm comparison average ranking chart.

The CEC2020 benchmark test suite is a new generation of test suite in the CEC series of test suites, containing 10 high-dimensional complex test functions. Compared with the CEC2014 test suite, its function structure is more complex, the number of local optimal solutions is larger, the search space is more rugged, and it has stronger nonlinearity and discontinuity, putting forward higher requirements for the global exploration capability, robustness and high-dimensional adaptability of the algorithm, and can more strictly test the performance of the improved algorithm. The 10 functions of the test suite are all multimodal functions, and the higher the dimensions, the more complex the function, which can effectively test the optimization performance of the 8 algorithms in high-dimensional complex scenarios, making up for the deficiency of the CEC2014 test suite in the high-dimensional scenario test. In this study, experiments were carried out on the 10 functions of the CEC2020 test suite in 30 dimensions. The experimental settings were consistent with the CEC2014 test suite. The 8 algorithms ran independently 30 times, various evaluation indicators were counted, and convergence curves were plotted. Table 2 shows the comparison of data results calculated by the 8 CEC2020 algorithms, Figure 5 shows the convergence curves of the 8 CEC2020 algorithms, Figure 6 shows the performance comparison data box of the CEC2020 algorithms, Figure 7 shows the radar chart of CEC2020 test set algorithm comparison, and Figure 8 shows the average ranking chart of CEC2020 test set algorithm comparison.

First, analysis of performance in high-dimensional scenarios. The CEC2020 test suite is characterized by high dimensionality and complexity, so this study focuses on comparing the optimization accuracy, convergence speed, and robustness of the 8 algorithms in high-dimensional environments. As the dimension increases, the search space expands drastically, increasing the difficulty of individual search. Traditional algorithms and some new algorithms are prone to issues such as rapid loss of population diversity and premature convergence, leading to significant attenuation of optimization performance. The accumulated difference foraging strategy of the CEGCRA can effectively maintain population diversity and prevent premature individual convergence. Meanwhile, the ergodicity of the piecewise chaotic map ensures that the initial population can fully cover the high-dimensional search space. Thus, in the 30-dimensional scenario, the CEGCRA should outperform the other 7 comparative algorithms in terms of optimization accuracy, convergence speed, and robustness.

The functions in the CEC2020 test suite exhibit strong randomness and complexity, which can effectively test the algorithm’s stability and anti-interference ability. This study focuses on comparing the standard deviations of the 8 algorithms to analyze the dispersion of algorithm results across multiple runs. By optimizing the position update mechanism and introducing chaotic randomness and accumulated difference information, the CEGCRA reduces the algorithm’s dependence on initial values and random factors. Therefore, its standard deviation should be significantly smaller than that of other comparative algorithms, especially the original GCRA, indicating that the CEGCRA has stronger operational stability and better anti-interference ability in complex scenarios.

Comprehensive experimental analysis of the CEC2014 and CEC2020 test suites leads to the following core conclusions: through the synergistic improvement of piecewise chaotic map initialization and the accumulated difference foraging strategy, the CEGCRA effectively compensates for the inherent defects of the original GCRA and is significantly superior to the original GCRA in terms of optimization accuracy, convergence speed, robustness, and high-dimensional adaptability. At the same time, compared with classical algorithms such as PSO and DE, as well as new algorithms such as the DBO, BKA, SSA, and FOX, the CEGCRA also demonstrates obvious advantages in comprehensive performance. This verifies the scientificity and effectiveness of the improvement strategy proposed in this paper and lays a theoretical foundation for subsequent engineering applications.

### 4.3. Engineering Application Experiments

The benchmark test suite experiments verify the theoretical optimization performance of the CEGCRA. To further test the practical application value of the algorithm, drawing on the engineering application design ideas, the CEGCRA was applied to two classical mechanical structure engineering constrained optimization problems of welded beam design and cantilever beam design. Both types of problems are multi-variable, multi-constraint and nonlinear optimization problems which widely exist in the field of mechanical design. The goal is to minimize manufacturing costs or structural weight on the premise of meeting constraint conditions such as structural strength, geometric dimensions and deflection, which have high requirements for the optimization accuracy, constraint handling capability and practicability of the algorithm, and can effectively test the practical engineering application potential of the CEGCRA.

In this engineering application experiment, the 8 comparative algorithms (CEGCRA, GCRA, PSO, DE, DBO, BKA, SSA, FOX) were retained for comparative experiments, and the experimental parameters were consistent with the benchmark test suite experiments to ensure the coherence and fairness of the experiments. The penalty function method was adopted for constraint condition processing, which converts the constraint conditions into penalty terms of the fitness function and the constrained optimization problem into an unconstrained optimization problem for easy solution by the algorithm.

#### 4.3.1. Welded Beam Design Problem

**Figure 9:**
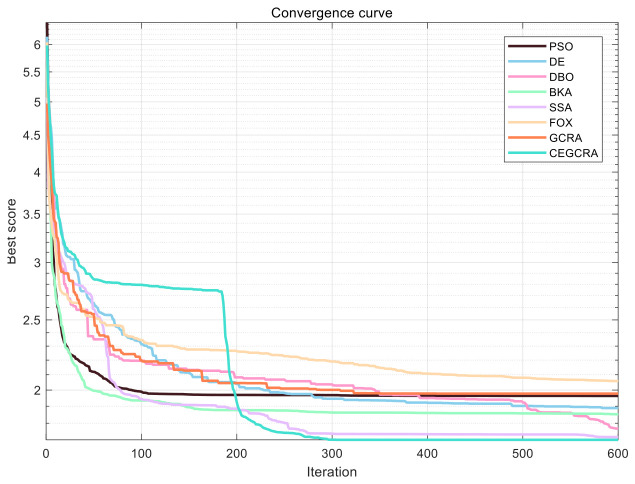
Comparison of optimization design algorithm results for welded beam.

Welded beam design is a classical constrained optimization problem in the field of mechanical manufacturing. Its core design goal is to minimize the manufacturing cost of the welded beam on the premise of meeting multiple constraint conditions such as shear stress, bending stress, buckling load, deflection and geometric dimensions, taking into account economy and structural reliability. The modeling of design variables, constraint conditions and objective function of the problem is as follows:

1. Design variables. The welded beam design includes 4 continuous design variables: weld thickness h ($X_{1}$), beam length l ($X_{2}$), beam height b ($X_{3}$) and steel plate thickness t ($X_{4}$). The search space boundary of the design variables is set as $0.125\leq X_{1}\leq 10$, $0.1\leq X_{2}\leq 10$, $0.1\leq X_{3}\leq 10$, $0.125\leq X_{4}\leq 10$ (unit: in), which is in line with the dimensional requirements of actual engineering design.
2. Objective function. The objective function is to minimize the manufacturing cost of the welded beam. Considering factors such as material cost and processing cost, the expression of the objective function is
  (11) $$\min f(X)=1.10471X_{1}^{2}X_{2}+0.04811X_{3}X_{4}(14.0+X_{2})$$
3. Constraint conditions. The welded beam design needs to meet 7 constraint conditions, covering shear stress, bending stress, buckling load, deflection, geometric dimensions, etc., as follows:
  (12) $$\left\{\begin{array}{l} \tau (X)\leq \tau_{\max}\\ \sigma (X)\leq \sigma_{\max}\\ \delta (X)\leq \delta_{\max}\\ X_{1}-X_{4}\leq 0\\ P-P_{c}(X)\leq 0\\ 0.125-X_{1}\leq 0\\ 0.1-X_{2}\leq 0\\ 0.1-X_{3}\leq 0\\ 0.125-X_{4}\leq 0 \end{array}\right.$$
  where $\tau \left(X\right)$ is the shear stress of the welded beam, $\tau_{max}=13,600$ psi (maximum allowable shear stress); $\sigma \left(X\right)$ is the bending stress of the welded beam, $\sigma_{max}=30,000$ psi (maximum allowable bending stress); $\delta \left(X\right)$ is the deflection of the welded beam, $\delta_{max}=0.25$ in (maximum allowable deflection); P=6000 lb (applied load); $P_{c}\left(X\right)$ is the buckling load of the welded beam, which needs to meet the buckling strength requirements. The remaining constraints are geometric dimension constraints to ensure that the design variables meet the requirements of actual engineering manufacturing. The comparison of optimization design algorithm results for welded beams is shown in Figure 9. The specific calculation results of the 8 algorithms are shown in Table 3.

By comparing the optimization results of the 8 algorithms in the welded beam design problem, the focus is on analyzing whether each algorithm can strictly meet all constraint conditions and the level of manufacturing cost after optimization. Due to its high optimization accuracy and constraint handling capability, the CEGCRA can accurately explore the optimal combination of design variables on the premise of meeting all constraint conditions. Therefore, the manufacturing cost after optimization should be significantly lower than that of the other 7 comparative algorithms, especially the original GCRA; at the same time, the CEGCRA has a faster convergence speed and can quickly find the optimal design scheme and improve the efficiency of engineering design. Through this experiment, the practicability and superiority of the CEGCRA in multi-constraint and nonlinear engineering optimization problems are verified, providing a better optimization scheme for welded beam design.

#### 4.3.2. Cantilever Beam Design Problem

Cantilever beam design is another classical constrained optimization problem in the field of mechanical structure design. Its core design goal is to minimize the structural weight of the cantilever beam on the premise of meeting the bending stress constraint, realize the structural lightweight design, and take into account structural strength and economy. The modeling of design variables, constraint conditions and objective function of the problem is as follows:

1. Design variables. The cantilever beam design includes 3 continuous design variables: beam width b ($X_{1}$), beam height h ($X_{2}$) and beam length L ($X_{3}$). The search space boundary of the design variables was set as $0.1\leq X_{1}\leq 10$, $0.1\leq X_{2}\leq 10$, $1\leq X_{3}\leq 20$ (unit: in), which is in line with the design dimension range of actual cantilever beams.
2. Objective function. The objective function is to minimize the weight of the cantilever beam. The cantilever beam is made of steel, with the steel density ρ=0.28 lb/in^3^. The expression of the objective function is
  (13) $$\min f(X)=\rho \times X_{1}\times X_{2}\times X_{3}$$
3. Constraint conditions. The core constraint of cantilever beam design is the bending stress constraint. The bending stress must not exceed the maximum allowable value, and the geometric dimension constraints must also be met, as follows:
  (14) $$\left\{\begin{array}{l} \sigma (X)\leq \sigma_{\max}\\ 0.1-X_{1}\leq 0\\ 0.1-X_{2}\leq 0\\ 1-X_{3}\leq 0 \end{array}\right.$$
  where $\sigma \left(X\right)$ is the bending stress of the cantilever beam, and its calculation formula is $\sigma \left(X\right)=\frac{6PL}{X_{1}X_{2}^{2}}$. P=1000 lb (applied load). $\sigma_{max}=30,000$ psi (maximum allowable bending stress). The remaining constraints are geometric dimension constraints to ensure that the design variables meet the requirements of actual manufacturing.

**Figure 10:**
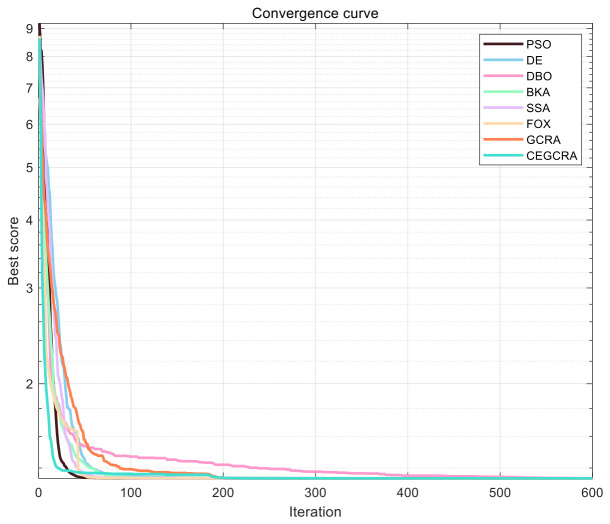
Comparison of optimization design algorithm results for cantilever beams.

The comparison of optimization design algorithm results for cantilever beams is shown in Figure 10. The specific calculation results of the 8 algorithms for cantilever beams are shown in Table 4.

By comparing the optimization results of the 8 algorithms in the cantilever beam design problem, the analysis focuses on whether each algorithm can satisfy the bending stress constraint and the level of structural weight after optimization. With its high optimization accuracy and strong local exploitation capability, the CEGCRA can identify the optimal combination of design variables under the premise of meeting the bending stress constraint so as to minimize the structural weight. Meanwhile, it possesses stronger robustness, with a smaller dispersion degree of results in multiple runs, which can provide a stable and reliable optimization scheme for engineering design. The expected experimental results are as follows: the weight of the cantilever beam optimized by the CEGCRA is significantly lower than that of the other 7 comparative algorithms, and its convergence speed is faster than that of most comparative algorithms. This verifies the practicability of the CEGCRA in structural lightweight engineering optimization problems and provides an efficient optimization method for cantilever beam design.

Through comprehensive experimental analysis of the CEC2014, CEC2020 benchmark test suites as well as the welded beam and cantilever beam engineering applications, the performance of the 8 comparative algorithms was comprehensively summarized and analyzed, with the focus on verifying the optimization performance and engineering application value of the CEGCRA. The following core conclusions are drawn: First, from the perspective of benchmark test suite experiments, the CEGCRA exhibits excellent comprehensive performance on test functions of different dimensions and types. Compared with the original GCRA, its optimization accuracy, convergence speed and robustness are significantly improved, which fully verifies the scientificity and effectiveness of the piecewise chaotic map initialization and accumulated difference foraging strategy. Compared with classical algorithms such as PSO and DE, the advantages of the CEGCRA are mainly reflected in high-dimensional scenarios and multimodal function tests, where it can effectively avoid premature convergence and improve optimization accuracy. Compared with new algorithms such as the DBO, BKA, SSA and FOX, CEGCRA has obvious advantages in comprehensive performance, especially in complex high-dimensional scenarios, indicating that it has strong competitiveness among similar new swarm intelligence algorithms. Second, from the perspective of engineering application experiments, the CEGCRA can effectively handle multi-constraint and nonlinear optimization problems in welded beam design and cantilever beam design, strictly meet all constraint conditions, and achieve the minimization of manufacturing cost or structural weight, with the optimization effect significantly superior to the other 7 comparative algorithms. This indicates that the CEGCRA not only has excellent theoretical optimization performance but also possesses strong practical engineering application capability, which can provide efficient and reliable optimization schemes for mechanical structure design and improve the economy and reliability of engineering design. Third, the improvement strategy of the CEGCRA has strong generality and expandability. The combination of the piecewise chaotic map and the accumulated difference foraging strategy can not only effectively improve the performance of the GCRA but also provide a reference idea for the improvement of other swarm intelligence algorithms. Meanwhile, the CEGCRA has simple parameter settings, is easy to implement, does not require complex parameter tuning, is convenient for engineering and technical personnel to apply in practice, and thus has strong engineering practicability.

## 5. Conclusions

To address the inherent shortcomings of the standard Greater Cane Rat Algorithm (GCRA) in solving complex optimization problems—including uneven population initialization distribution, rapid loss of population diversity, proneness to falling into local optima, and unbalanced exploration and exploitation—a Chaos-Integrated Difference-Enhanced Greater Cane Rat Algorithm (CEGCRA) is proposed. Through targeted improvement strategies, the comprehensive optimization performance of the algorithm is fully enhanced, and the effectiveness and practicability of the algorithm are verified through benchmark test suite experiments and engineering application tests.

The core research work and innovations of this paper are mainly reflected in two aspects: first, the piecewise chaotic map is introduced to optimize the population initialization process, replacing the random initialization method of the original GCRA. Leveraging the excellent ergodicity and uniform distribution characteristics of the piecewise chaotic map, the initial population can fully cover the search space, improve the quality of initial solutions, and lay a solid foundation for the global optimization of the algorithm. Second, an accumulated difference foraging strategy is designed, which integrates the position difference and fitness difference information during the individual iteration process, incorporates historical search information into the position update mechanism, effectively maintains population diversity, prevents premature convergence of individuals, and strengthens the algorithm’s ability to escape local optima. In addition, the overall execution process of the CEGCRA is improved, with optimized boundary processing and difference attenuation mechanisms, which enhance the stability and robustness of the algorithm and enable it to adapt to more complex optimization scenarios. Experimental results demonstrate that the comprehensive performance of the CEGCRA on the benchmark test suite is significantly superior to that of the other seven comparative algorithms, and it can effectively handle complex optimization scenarios such as low-dimensional, high-dimensional, multi-extremum, and strong nonlinearity. In engineering applications, it can strictly meet constraint conditions, optimize design objectives, and outperform the original GCRA and other comparative algorithms significantly. This verifies the scientificity and effectiveness of the improvement strategy proposed in this paper and also proves that the CEGCRA has excellent theoretical value and engineering application potential.

Although the CEGCRA has demonstrated excellent optimization performance in this experiment, there is still room for further optimization and expansion. Combined with the current development trend of swarm intelligence algorithms, future research directions will focus on the following three aspects: First, an adaptive parameter adjustment mechanism will be introduced to dynamically regulate core parameters such as chaos parameters, inertia weight, and stage switching probability, adaptively adjusting parameter values according to the iteration process and population state to further improve the algorithm’s adaptability and optimization performance. Second, the CEGCRA will be extended to the field of multi-objective optimization, combining it with multi-objective optimization methods such as non-dominated sorting and crowding degree calculation to solve common multi-objective collaborative optimization problems in engineering, thereby further expanding the algorithm’s application scope. Third, the CEGCRA will be applied to more practical engineering scenarios, such as microgrid optimization, path planning, image processing, and parameter tuning, the algorithm’s application potential will be further explored, and the algorithm’s constraint handling mechanism will be optimized according to the characteristics of different engineering scenarios to enhance the algorithm’s engineering adaptability.

## Figures and Tables

**Table 1:** The result of standard functions of CEC2014 for different algorithms.

		PSO	DE	DBO	BKA	SSA	FOX	GCRA	CEGCRA
F1	min	6.22 × 10^6^	5.61 × 10^7^	2.84 × 10^8^	3.68 × 10^6^	6.03 × 10^5^	1.71 × 10^6^	3.23 × 10^6^	2.27 × 10^6^
F1	std	3.43 × 10^7^	2.86 × 10^7^	1.16 × 10^8^	2.46 × 10^8^	1.01 × 10^6^	8.88 × 10^5^	6.85 × 10^6^	2.15 × 10^6^
F1	avg	2.98 × 10^7^	1.12 × 10^8^	5.69 × 10^8^	1.14 × 10^8^	1.73 × 10^6^	3.46 × 10^6^	1.35 × 10^7^	5.94 × 10^6^
F1	median	2.50 × 10^7^	1.10 × 10^8^	6.08 × 10^8^	1.39 × 10^7^	1.39 × 10^6^	3.61 × 10^6^	1.48 × 10^7^	5.57 × 10^6^
F2	min	1.88 × 10^6^	7.56 × 10^2^	3.91 × 10^10^	4.43 × 10^8^	2.04 × 10^2^	8.65 × 10^3^	9.67 × 10^3^	3.46 × 10^4^
F2	std	1.41 × 10^9^	4.29 × 10^3^	4.57 × 10^9^	1.74 × 10^10^	1.10 × 10^3^	2.43 × 10^3^	2.52 × 10^5^	1.81 × 10^5^
F2	avg	1.25 × 10^9^	3.00 × 10^3^	4.65 × 10^10^	1.04 × 10^10^	1.38 × 10^3^	1.21 × 10^4^	3.17 × 10^5^	1.91 × 10^5^
F2	median	8.64 × 10^8^	1.50 × 10^3^	4.71 × 10^10^	2.69 × 10^9^	8.65 × 10^2^	1.22 × 10^4^	2.62 × 10^5^	1.51 × 10^5^
F3	min	5.90 × 10^3^	7.89 × 10^2^	4.13 × 10^4^	1.24 × 10^4^	5.01 × 10^2^	4.67 × 10^4^	5.40 × 10^3^	4.58 × 10^3^
F3	std	1.22 × 10^4^	2.52 × 10^3^	1.10 × 10^4^	9.20 × 10^3^	4.30 × 10^3^	5.07 × 10^4^	1.23 × 10^4^	1.06 × 10^4^
F3	avg	2.22 × 10^4^	3.35 × 10^3^	6.54 × 10^4^	2.37 × 10^4^	4.26 × 10^3^	1.24 × 10^5^	2.41 × 10^4^	1.98 × 10^4^
F3	median	2.01 × 10^4^	3.06 × 10^3^	6.98 × 10^4^	2.22 × 10^4^	2.28 × 10^3^	1.33 × 10^5^	2.38 × 10^4^	1.69 × 10^4^
F4	min	4.83 × 10^2^	5.41 × 10^2^	1.65 × 10^3^	5.64 × 10^2^	4.54 × 10^2^	4.66 × 10^2^	5.13 × 10^2^	4.58 × 10^2^
F4	std	1.08 × 10^2^	1.73 × 10^1^	1.18 × 10^3^	3.28 × 10^3^	3.64 × 10^1^	2.67 × 10^1^	1.61 × 10^1^	2.36 × 10^1^
F4	avg	6.60 × 10^2^	5.64 × 10^2^	3.37 × 10^3^	1.82 × 10^3^	5.02 × 10^2^	4.97 × 10^2^	5.41 × 10^2^	5.05 × 10^2^
F4	median	6.34 × 10^2^	5.59 × 10^2^	3.20 × 10^3^	6.75 × 10^2^	4.83 × 10^2^	4.83 × 10^2^	5.42 × 10^2^	5.04 × 10^2^
F5	min	5.20 × 10^2^	5.21 × 10^2^	5.21 × 10^2^	5.20 × 10^2^	5.20 × 10^2^	5.20 × 10^2^	5.21 × 10^2^	5.21 × 10^2^
F5	std	2.73 × 10^−1^	5.69 × 10^−2^	7.89 × 10^−2^	2.17 × 10^−1^	2.56 × 10^−1^	2.91 × 10^−3^	4.97 × 10^−2^	4.51 × 10^−2^
F5	avg	5.20 × 10^2^	5.21 × 10^2^	5.21 × 10^2^	5.21 × 10^2^	5.20 × 10^2^	5.20 × 10^2^	5.21 × 10^2^	5.21 × 10^2^
F5	median	5.20 × 10^2^	5.21 × 10^2^	5.21 × 10^2^	5.21 × 10^2^	5.20 × 10^2^	5.20 × 10^2^	5.21 × 10^2^	5.21 × 10^2^
F6	min	6.15 × 10^2^	6.27 × 10^2^	6.30 × 10^2^	6.28 × 10^2^	6.21 × 10^2^	6.33 × 10^2^	6.14 × 10^2^	6.13 × 10^2^
F6	std	3.05 × 10^0^	1.48 × 10^0^	1.60 × 10^0^	2.40 × 10^0^	3.22 × 10^0^	2.49 × 10^0^	2.31 × 10^0^	3.13 × 10^0^
F6	avg	6.25 × 10^2^	6.30 × 10^2^	6.32 × 10^2^	6.31 × 10^2^	6.28 × 10^2^	6.39 × 10^2^	6.18 × 10^2^	6.18 × 10^2^
F6	median	6.25 × 10^2^	6.31 × 10^2^	6.32 × 10^2^	6.31 × 10^2^	6.28 × 10^2^	6.39 × 10^2^	6.19 × 10^2^	6.18 × 10^2^
F7	min	7.01 × 10^2^	7.00 × 10^2^	1.03 × 10^3^	7.01 × 10^2^	7.00 × 10^2^	7.00 × 10^2^	7.00 × 10^2^	7.00 × 10^2^
F7	std	2.23 × 10^1^	4.29 × 10^−2^	7.12 × 10^1^	1.25 × 10^2^	1.49 × 10^−2^	1.51 × 10^−2^	1.80 × 10^−1^	1.54 × 10^−1^
F7	avg	7.24 × 10^2^	7.00 × 10^2^	1.17 × 10^3^	7.67 × 10^2^	7.00 × 10^2^	7.00 × 10^2^	7.00 × 10^2^	7.00 × 10^2^
F7	median	7.21 × 10^2^	7.00 × 10^2^	1.18 × 10^3^	7.38 × 10^2^	7.00 × 10^2^	7.00 × 10^2^	7.00 × 10^2^	7.00 × 10^2^
F8	min	8.85 × 10^2^	8.49 × 10^2^	1.03 × 10^3^	9.21 × 10^2^	8.94 × 10^2^	9.50 × 10^2^	8.53 × 10^2^	8.48 × 10^2^
F8	std	3.47 × 10^1^	3.83 × 10^0^	1.96 × 10^1^	3.29 × 10^1^	2.66 × 10^1^	5.78 × 10^1^	1.33 × 10^1^	1.96 × 10^1^
F8	avg	9.39 × 10^2^	8.56 × 10^2^	1.07 × 10^3^	9.71 × 10^2^	9.31 × 10^2^	1.02 × 10^3^	8.75 × 10^2^	8.78 × 10^2^
F8	median	9.32 × 10^2^	8.57 × 10^2^	1.06 × 10^3^	9.63 × 10^2^	9.29 × 10^2^	9.90 × 10^2^	8.73 × 10^2^	8.75 × 10^2^
F9	min	1.00 × 10^3^	1.06 × 10^3^	1.14 × 10^3^	1.03 × 10^3^	1.05 × 10^3^	1.08 × 10^3^	9.77 × 10^2^	9.72 × 10^2^
F9	std	3.06 × 10^1^	1.21 × 10^1^	2.15 × 10^1^	3.86 × 10^1^	1.57 × 10^1^	5.03 × 10^0^	1.48 × 10^1^	2.30 × 10^1^
F9	avg	1.05 × 10^3^	1.08 × 10^3^	1.17 × 10^3^	1.10 × 10^3^	1.09 × 10^3^	1.09 × 10^3^	9.98 × 10^2^	1.01 × 10^3^
F9	median	1.03 × 10^3^	1.08 × 10^3^	1.18 × 10^3^	1.09 × 10^3^	1.09 × 10^3^	1.09 × 10^3^	9.96 × 10^2^	1.00 × 10^3^
F10	min	2.78 × 10^3^	1.52 × 10^3^	5.83 × 10^3^	3.90 × 10^3^	2.87 × 10^3^	4.24 × 10^3^	1.81 × 10^3^	1.39 × 10^3^
F10	std	6.98 × 10^2^	2.63 × 10^2^	5.43 × 10^2^	9.38 × 10^2^	5.79 × 10^2^	7.85 × 10^2^	4.27 × 10^2^	3.56 × 10^2^
F10	avg	4.02 × 10^3^	2.17 × 10^3^	6.66 × 10^3^	4.92 × 10^3^	3.88 × 10^3^	5.49 × 10^3^	2.71 × 10^3^	2.05 × 10^3^
F10	median	3.98 × 10^3^	2.15 × 10^3^	6.61 × 10^3^	4.76 × 10^3^	3.89 × 10^3^	5.40 × 10^3^	2.68 × 10^3^	2.03 × 10^3^
F11	min	3.64 × 10^3^	6.27 × 10^3^	6.48 × 10^3^	4.51 × 10^3^	3.81 × 10^3^	4.33 × 10^3^	2.88 × 10^3^	2.35 × 10^3^
F11	std	7.88 × 10^2^	2.97 × 10^2^	7.15 × 10^2^	1.15 × 10^3^	7.85 × 10^2^	7.63 × 10^2^	4.24 × 10^2^	5.21 × 10^2^
F11	avg	4.89 × 10^3^	7.15 × 10^3^	8.21 × 10^3^	5.46 × 10^3^	5.02 × 10^3^	5.74 × 10^3^	3.53 × 10^3^	3.45 × 10^3^
F11	median	4.86 × 10^3^	7.17 × 10^3^	8.40 × 10^3^	4.98 × 10^3^	4.93 × 10^3^	5.60 × 10^3^	3.49 × 10^3^	3.46 × 10^3^
F12	min	1.20 × 10^3^	1.20 × 10^3^	1.20 × 10^3^	1.20 × 10^3^	1.20 × 10^3^	1.20 × 10^3^	1.20 × 10^3^	1.20 × 10^3^
F12	std	1.90 × 10^−1^	2.38 × 10^−1^	4.57 × 10^−1^	2.32 × 10^−1^	4.20 × 10^−1^	1.42 × 10^−1^	5.39 × 10^−1^	1.16 × 10^0^
F12	avg	1.20 × 10^3^	1.20 × 10^3^	1.20 × 10^3^	1.20 × 10^3^	1.20 × 10^3^	1.20 × 10^3^	1.20 × 10^3^	1.20 × 10^3^
F12	median	1.20 × 10^3^	1.20 × 10^3^	1.20 × 10^3^	1.20 × 10^3^	1.20 × 10^3^	1.20 × 10^3^	1.20 × 10^3^	1.20 × 10^3^
F13	min	1.30 × 10^3^	1.30 × 10^3^	1.30 × 10^3^	1.30 × 10^3^	1.30 × 10^3^	1.30 × 10^3^	1.30 × 10^3^	1.30 × 10^3^
F13	std	4.79 × 10^−1^	6.47 × 10^−2^	3.83 × 10^−1^	1.81 × 10^0^	1.14 × 10^−1^	8.76 × 10^−2^	8.92 × 10^−2^	5.48 × 10^−2^
F13	avg	1.30 × 10^3^	1.30 × 10^3^	1.31 × 10^3^	1.30 × 10^3^	1.30 × 10^3^	1.30 × 10^3^	1.30 × 10^3^	1.30 × 10^3^
F13	median	1.30 × 10^3^	1.30 × 10^3^	1.31 × 10^3^	1.30 × 10^3^	1.30 × 10^3^	1.30 × 10^3^	1.30 × 10^3^	1.30 × 10^3^
F14	min	1.40 × 10^3^	1.40 × 10^3^	1.48 × 10^3^	1.40 × 10^3^	1.40 × 10^3^	1.40 × 10^3^	1.40 × 10^3^	1.40 × 10^3^
F14	std	1.15 × 10^1^	8.03 × 10^−2^	3.02 × 10^1^	6.80 × 10^1^	1.19 × 10^−1^	4.60 × 10^−2^	1.11 × 10^−1^	3.46 × 10^−2^
F14	avg	1.41 × 10^3^	1.40 × 10^3^	1.55 × 10^3^	1.44 × 10^3^	1.40 × 10^3^	1.40 × 10^3^	1.40 × 10^3^	1.40 × 10^3^
F14	median	1.40 × 10^3^	1.40 × 10^3^	1.56 × 10^3^	1.41 × 10^3^	1.40 × 10^3^	1.40 × 10^3^	1.40 × 10^3^	1.40 × 10^3^
F15	min	1.52 × 10^3^	1.52 × 10^3^	4.26 × 10^3^	1.54 × 10^3^	1.51 × 10^3^	1.52 × 10^3^	1.51 × 10^3^	1.51 × 10^3^
F15	std	9.60 × 10^1^	1.14 × 10^0^	2.32 × 10^4^	3.73 × 10^4^	6.71 × 10^0^	8.63 × 10^0^	3.89 × 10^0^	4.53 × 10^0^
F15	avg	1.57 × 10^3^	1.52 × 10^3^	6.23 × 10^4^	1.86 × 10^4^	1.52 × 10^3^	1.54 × 10^3^	1.52 × 10^3^	1.51 × 10^3^
F15	median	1.55 × 10^3^	1.52 × 10^3^	6.49 × 10^4^	1.85 × 10^3^	1.52 × 10^3^	1.54 × 10^3^	1.52 × 10^3^	1.52 × 10^3^
F16	min	1.61 × 10^3^	1.61 × 10^3^	1.61 × 10^3^	1.61 × 10^3^	1.61 × 10^3^	1.61 × 10^3^	1.61 × 10^3^	1.61 × 10^3^
F16	std	5.57 × 10^−1^	2.29 × 10^−1^	2.65 × 10^−1^	4.19 × 10^−1^	4.91 × 10^−1^	2.29 × 10^−1^	4.14 × 10^−1^	4.97 × 10^−1^
F16	avg	1.61 × 10^3^	1.61 × 10^3^	1.61 × 10^3^	1.61 × 10^3^	1.61 × 10^3^	1.61 × 10^3^	1.61 × 10^3^	1.61 × 10^3^
F16	median	1.61 × 10^3^	1.61 × 10^3^	1.61 × 10^3^	1.61 × 10^3^	1.61 × 10^3^	1.61 × 10^3^	1.61 × 10^3^	1.61 × 10^3^
F17	min	6.74 × 10^4^	3.38 × 10^6^	9.65 × 10^6^	1.41 × 10^4^	7.40 × 10^4^	4.68 × 10^4^	1.46 × 10^5^	1.41 × 10^5^
F17	std	1.04 × 10^6^	2.08 × 10^6^	1.35 × 10^7^	1.47 × 10^6^	3.04 × 10^5^	2.45 × 10^5^	9.23 × 10^5^	3.69 × 10^5^
F17	avg	8.84 × 10^5^	6.37 × 10^6^	2.72 × 10^7^	7.38 × 10^5^	4.59 × 10^5^	3.19 × 10^5^	8.21 × 10^5^	5.89 × 10^5^
F17	median	4.46 × 10^5^	6.46 × 10^6^	2.64 × 10^7^	1.92 × 10^5^	3.80 × 10^5^	2.30 × 10^5^	5.86 × 10^5^	5.16 × 10^5^
F18	min	2.20 × 10^3^	7.32 × 10^4^	8.97 × 10^7^	7.12 × 10^3^	2.20 × 10^3^	2.06 × 10^3^	2.25 × 10^3^	1.95 × 10^3^
F18	std	5.99 × 10^3^	2.97 × 10^5^	2.59 × 10^8^	2.26 × 10^8^	6.31 × 10^3^	2.45 × 10^3^	4.32 × 10^3^	1.60 × 10^3^
F18	avg	6.95 × 10^3^	4.14 × 10^5^	2.70 × 10^8^	7.03 × 10^7^	7.97 × 10^3^	3.36 × 10^3^	6.27 × 10^3^	3.14 × 10^3^
F18	median	4.62 × 10^3^	3.44 × 10^5^	2.31 × 10^8^	2.76 × 10^4^	5.94 × 10^3^	2.64 × 10^3^	4.76 × 10^3^	2.36 × 10^3^
F19	min	1.91 × 10^3^	1.91 × 10^3^	2.05 × 10^3^	1.92 × 10^3^	1.91 × 10^3^	1.91 × 10^3^	1.91 × 10^3^	1.91 × 10^3^
F19	std	2.58 × 10^1^	3.68 × 10^0^	6.72 × 10^1^	1.04 × 10^2^	1.67 × 10^1^	2.66 × 10^1^	2.34 × 10^1^	1.84 × 10^0^
F19	avg	1.93 × 10^3^	1.91 × 10^3^	2.12 × 10^3^	1.99 × 10^3^	1.92 × 10^3^	1.93 × 10^3^	1.92 × 10^3^	1.91 × 10^3^
F19	median	1.92 × 10^3^	1.91 × 10^3^	2.09 × 10^3^	1.97 × 10^3^	1.91 × 10^3^	1.92 × 10^3^	1.91 × 10^3^	1.91 × 10^3^
F20	min	7.85 × 10^3^	4.26 × 10^3^	2.72 × 10^4^	2.68 × 10^3^	5.52 × 10^3^	1.85 × 10^4^	8.25 × 10^3^	3.32 × 10^3^
F20	std	1.14 × 10^4^	5.90 × 10^3^	3.04 × 10^4^	3.55 × 10^4^	9.37 × 10^3^	2.08 × 10^4^	9.20 × 10^3^	4.85 × 10^3^
F20	avg	2.45 × 10^4^	1.36 × 10^4^	6.65 × 10^4^	1.85 × 10^4^	1.67 × 10^4^	4.94 × 10^4^	2.02 × 10^4^	8.27 × 10^3^
F20	median	2.51 × 10^4^	1.32 × 10^4^	6.01 × 10^4^	9.32 × 10^3^	1.34 × 10^4^	5.12 × 10^4^	1.97 × 10^4^	6.55 × 10^3^
F21	min	1.69 × 10^4^	4.12 × 10^5^	6.97 × 10^5^	1.57 × 10^4^	4.97 × 10^4^	9.73 × 10^3^	3.93 × 10^4^	2.64 × 10^4^
F21	std	1.34 × 10^5^	4.91 × 10^5^	4.36 × 10^6^	7.96 × 10^5^	1.43 × 10^5^	1.71 × 10^5^	2.73 × 10^5^	1.17 × 10^5^
F21	avg	1.71 × 10^5^	1.33 × 10^6^	4.69 × 10^6^	3.90 × 10^5^	2.56 × 10^5^	2.18 × 10^5^	2.65 × 10^5^	1.69 × 10^5^
F21	median	1.34 × 10^5^	1.36 × 10^6^	3.13 × 10^6^	6.88 × 10^4^	2.33 × 10^5^	1.52 × 10^5^	1.59 × 10^5^	1.54 × 10^5^
F22	min	2.44 × 10^3^	2.38 × 10^3^	2.58 × 10^3^	2.48 × 10^3^	2.64 × 10^3^	2.69 × 10^3^	2.50 × 10^3^	2.55 × 10^3^
F22	std	2.46 × 10^2^	1.40 × 10^2^	3.22 × 10^2^	2.46 × 10^2^	2.00 × 10^2^	3.55 × 10^2^	1.66 × 10^2^	1.62 × 10^2^
F22	avg	2.85 × 10^3^	2.63 × 10^3^	3.33 × 10^3^	2.88 × 10^3^	3.00 × 10^3^	3.35 × 10^3^	2.74 × 10^3^	2.76 × 10^3^
F22	median	2.91 × 10^3^	2.63 × 10^3^	3.34 × 10^3^	2.82 × 10^3^	3.03 × 10^3^	3.41 × 10^3^	2.71 × 10^3^	2.73 × 10^3^
F23	min	2.62 × 10^3^	2.62 × 10^3^	2.50 × 10^3^	2.50 × 10^3^	2.50 × 10^3^	2.50 × 10^3^	2.50 × 10^3^	2.50 × 10^3^
F23	std	1.44 × 10^1^	1.03 × 10^−2^	1.15 × 10^2^	0	0	0	4.55 × 10^1^	0
F23	avg	2.63 × 10^3^	2.62 × 10^3^	2.56 × 10^3^	2.50 × 10^3^	2.50 × 10^3^	2.50 × 10^3^	2.54 × 10^3^	2.50 × 10^3^
F23	median	2.63 × 10^3^	2.62 × 10^3^	2.50 × 10^3^	2.50 × 10^3^	2.50 × 10^3^	2.50 × 10^3^	2.51 × 10^3^	2.50 × 10^3^
F24	min	2.63 × 10^3^	2.63 × 10^3^	2.60 × 10^3^	2.60 × 10^3^	2.60 × 10^3^	2.60 × 10^3^	2.60 × 10^3^	2.60 × 10^3^
F24	std	7.33 × 10^0^	1.43 × 10^0^	2.30 × 10^−4^	0	1.20 × 10^−5^	0	4.03 × 10^0^	0
F24	avg	2.65 × 10^3^	2.63 × 10^3^	2.60 × 10^3^	2.60 × 10^3^	2.60 × 10^3^	2.60 × 10^3^	2.60 × 10^3^	2.60 × 10^3^
F24	median	2.65 × 10^3^	2.63 × 10^3^	2.60 × 10^3^	2.60 × 10^3^	2.60 × 10^3^	2.60 × 10^3^	2.60 × 10^3^	2.60 × 10^3^
F25	min	2.71 × 10^3^	2.72 × 10^3^	2.70 × 10^3^	2.70 × 10^3^	2.70 × 10^3^	2.70 × 10^3^	2.70 × 10^3^	2.70 × 10^3^
F25	std	5.04 × 10^0^	4.03 × 10^0^	0	0	0	0	9.56 × 10^−7^	0
F25	avg	2.72 × 10^3^	2.72 × 10^3^	2.70 × 10^3^	2.70 × 10^3^	2.70 × 10^3^	2.70 × 10^3^	2.70 × 10^3^	2.70 × 10^3^
F25	median	2.72 × 10^3^	2.72 × 10^3^	2.70 × 10^3^	2.70 × 10^3^	2.70 × 10^3^	2.70 × 10^3^	2.70 × 10^3^	2.70 × 10^3^
F26	min	2.70 × 10^3^	2.70 × 10^3^	2.70 × 10^3^	2.70 × 10^3^	2.70 × 10^3^	2.70 × 10^3^	2.70 × 10^3^	2.70 × 10^3^
F26	std	6.39 × 10^1^	6.43 × 10^−2^	4.48 × 10^1^	1.03 × 10^0^	4.42 × 10^1^	2.23 × 10^1^	3.65 × 10^1^	2.15 × 10^1^
F26	avg	2.79 × 10^3^	2.70 × 10^3^	2.73 × 10^3^	2.70 × 10^3^	2.73 × 10^3^	2.80 × 10^3^	2.72 × 10^3^	2.71 × 10^3^
F26	median	2.80 × 10^3^	2.70 × 10^3^	2.71 × 10^3^	2.70 × 10^3^	2.70 × 10^3^	2.80 × 10^3^	2.70 × 10^3^	2.70 × 10^3^
F27	min	3.11 × 10^3^	3.28 × 10^3^	3.17 × 10^3^	2.90 × 10^3^	2.90 × 10^3^	2.90 × 10^3^	2.90 × 10^3^	2.90 × 10^3^
F27	std	2.35 × 10^2^	1.31 × 10^2^	6.33 × 10^1^	0	0	0	2.60 × 10^2^	0
F27	avg	3.60 × 10^3^	3.52 × 10^3^	3.27 × 10^3^	2.90 × 10^3^	2.90 × 10^3^	2.90 × 10^3^	3.09 × 10^3^	2.90 × 10^3^
F27	median	3.67 × 10^3^	3.49 × 10^3^	3.26 × 10^3^	2.90 × 10^3^	2.90 × 10^3^	2.90 × 10^3^	2.96 × 10^3^	2.90 × 10^3^
F28	min	4.00 × 10^3^	3.67 × 10^3^	3.00 × 10^3^	3.00 × 10^3^	3.00 × 10^3^	3.00 × 10^3^	3.00 × 10^3^	3.00 × 10^3^
F28	std	8.96 × 10^2^	3.67 × 10^1^	7.06 × 10^2^	0	0	0	4.07 × 10^2^	0
F28	avg	5.36 × 10^3^	3.74 × 10^3^	5.34 × 10^3^	3.00 × 10^3^	3.00 × 10^3^	3.00 × 10^3^	3.25 × 10^3^	3.00 × 10^3^
F28	median	5.14 × 10^3^	3.75 × 10^3^	5.37 × 10^3^	3.00 × 10^3^	3.00 × 10^3^	3.00 × 10^3^	3.01 × 10^3^	3.00 × 10^3^
F29	min	4.46 × 10^3^	4.17 × 10^3^	3.10 × 10^3^	3.10 × 10^3^	3.10 × 10^3^	3.10 × 10^3^	4.26 × 10^3^	4.31 × 10^3^
F29	std	6.75 × 10^6^	1.41 × 10^5^	3.96 × 10^7^	6.08 × 10^3^	0	0	1.99 × 10^6^	2.15 × 10^6^
F29	avg	3.34 × 10^6^	3.93 × 10^4^	5.22 × 10^7^	4.46 × 10^3^	3.10 × 10^3^	3.10 × 10^3^	4.53 × 10^5^	4.86 × 10^5^
F29	median	8.12 × 10^4^	7.86 × 10^3^	4.91 × 10^7^	3.10 × 10^3^	3.10 × 10^3^	3.10 × 10^3^	5.31 × 10^3^	5.11 × 10^3^
F30	min	6.98 × 10^3^	7.45 × 10^3^	2.25 × 10^5^	7.77 × 10^3^	3.20 × 10^3^	3.20 × 10^3^	5.78 × 10^3^	5.73 × 10^3^
F30	std	2.09 × 10^4^	2.42 × 10^3^	3.59 × 10^5^	2.68 × 10^4^	1.33 × 10^−6^	0	4.55 × 10^3^	4.09 × 10^3^
F30	avg	2.03 × 10^4^	1.37 × 10^4^	6.29 × 10^5^	2.51 × 10^4^	3.20 × 10^3^	3.20 × 10^3^	9.10 × 10^3^	9.07 × 10^3^
F30	median	1.24 × 10^4^	1.34 × 10^4^	5.63 × 10^5^	1.62 × 10^4^	3.20 × 10^3^	3.20 × 10^3^	7.54 × 10^3^	7.78 × 10^3^

**Table 2:** The result of standard functions of CEC2020 for different algorithms.

		PSO	DE	DBO	BKA	SSA	FOX	GCRA	CEGCRA
F1	min	9.09 × 10^6^	1.84 × 10^4^	2.25 × 10^10^	2.86 × 10^8^	1.02 × 10^2^	3.64 × 10^3^	2.87 × 10^4^	2.22 × 10^4^
F1	std	7.56 × 10^8^	2.96 × 10^4^	2.97 × 10^9^	1.26 × 10^10^	7.47 × 10^3^	1.74 × 10^3^	9.22 × 10^4^	2.20 × 10^5^
F1	avg	6.79 × 10^8^	5.41 × 10^4^	2.89 × 10^10^	7.26 × 10^9^	5.32 × 10^3^	6.00 × 10^3^	1.78 × 10^5^	2.34 × 10^5^
F1	median	1.43 × 10^8^	4.83 × 10^4^	2.89 × 10^10^	2.81 × 10^9^	1.40 × 10^3^	6.49 × 10^3^	1.61 × 10^5^	1.61 × 10^5^
F2	min	3.14 × 10^3^	6.63 × 10^3^	6.44 × 10^3^	3.65 × 10^3^	3.85 × 10^3^	4.27 × 10^3^	2.60 × 10^3^	2.99 × 10^3^
F2	std	756.00	337.00	728.00	1.06 × 10^3^	831.00	557.00	451.00	543.00
F2	avg	4.93 × 10^3^	7.24 × 10^3^	8.12 × 10^3^	5.21 × 10^3^	5.23 × 10^3^	5.40 × 10^3^	3.58 × 10^3^	3.85 × 10^3^
F2	median	5.12 × 10^3^	7.18 × 10^3^	8.42 × 10^3^	5.17 × 10^3^	5.22 × 10^3^	5.42 × 10^3^	3.58 × 10^3^	3.79 × 10^3^
F3	min	839.00	898.00	1.14 × 10^3^	1.13 × 10^3^	1.07 × 10^3^	1.26 × 10^3^	822.00	846.00
F3	std	83.10	11.20	42.10	73.20	86.80	23.80	29.80	32.70
F3	avg	944.00	915.00	1.21 × 10^3^	1.26 × 10^3^	1.23 × 10^3^	1.32 × 10^3^	870.00	897.00
F3	median	922.00	916.00	1.20 × 10^3^	1.25 × 10^3^	1.21 × 10^3^	1.33 × 10^3^	866.00	891.00
F4	min	1.92 × 10^3^	1.91 × 10^3^	1.43 × 10^5^	1.97 × 10^3^	1.91 × 10^3^	1.93 × 10^3^	1.91 × 10^3^	1.91 × 10^3^
F4	std	5.43 × 10^3^	1.65	7.13 × 10^5^	9.21 × 10^5^	8.12	9.46	3.97	4.88
F4	avg	4.17 × 10^3^	1.92 × 10^3^	1.08 × 10^6^	2.10 × 10^5^	1.92 × 10^3^	1.94 × 10^3^	1.92 × 10^3^	1.91 × 10^3^
F4	median	2.00 × 10^3^	1.92 × 10^3^	1.05 × 10^6^	2.54 × 10^3^	1.92 × 10^3^	1.94 × 10^3^	1.92 × 10^3^	1.91 × 10^3^
F5	min	2.37 × 10^4^	3.10 × 10^6^	3.10 × 10^6^	5.67 × 10^4^	5.18 × 10^4^	8.86 × 10^4^	1.11 × 10^5^	2.22 × 10^5^
F5	std	6.31 × 10^5^	1.93 × 10^6^	1.43 × 10^7^	1.90 × 10^5^	4.88 × 10^5^	1.98 × 10^5^	5.67 × 10^5^	2.64 × 10^5^
F5	avg	6.54 × 10^5^	5.71 × 10^6^	2.25 × 10^7^	2.19 × 10^5^	7.52 × 10^5^	3.01 × 10^5^	7.77 × 10^5^	5.82 × 10^5^
F5	median	4.24 × 10^5^	5.47 × 10^6^	2.11 × 10^7^	1.66 × 10^5^	6.24 × 10^5^	2.12 × 10^5^	5.70 × 10^5^	5.21 × 10^5^
F6	min	1.92 × 10^3^	2.45 × 10^3^	2.79 × 10^3^	2.18 × 10^3^	1.81 × 10^3^	2.33 × 10^3^	1.71 × 10^3^	1.67 × 10^3^
F6	std	307.00	183.00	359.00	680.00	241.00	459.00	232.00	218.00
F6	avg	2.35 × 10^3^	2.82 × 10^3^	3.38 × 10^3^	2.90 × 10^3^	2.37 × 10^3^	3.33 × 10^3^	2.20 × 10^3^	2.09 × 10^3^
F6	median	2.33 × 10^3^	2.78 × 10^3^	3.31 × 10^3^	2.67 × 10^3^	2.34 × 10^3^	3.26 × 10^3^	2.23 × 10^3^	2.06 × 10^3^
F7	min	3.25 × 10^4^	3.76 × 10^5^	1.70 × 10^5^	1.86 × 10^4^	1.89 × 10^4^	4.89 × 10^4^	7.26 × 10^4^	1.97 × 10^4^
F7	std	2.61 × 10^5^	5.21 × 10^5^	3.01 × 10^6^	4.27 × 10^5^	1.67 × 10^5^	1.16 × 10^5^	2.36 × 10^5^	1.47 × 10^5^
F7	avg	1.74 × 10^5^	1.14 × 10^6^	3.93 × 10^6^	1.86 × 10^5^	2.71 × 10^5^	1.73 × 10^5^	2.68 × 10^5^	1.65 × 10^5^
F7	median	8.00 × 10^4^	1.11 × 10^6^	3.45 × 10^6^	5.83 × 10^4^	2.43 × 10^5^	1.32 × 10^5^	1.76 × 10^5^	1.35 × 10^5^
F8	min	2.35 × 10^3^	4.05 × 10^3^	4.72 × 10^3^	3.10 × 10^3^	2.30 × 10^3^	6.40 × 10^3^	2.31 × 10^3^	2.30 × 10^3^
F8	std	2.11 × 10^3^	1.63 × 10^3^	766.00	1.68 × 10^3^	1.94 × 10^3^	630.00	1.43 × 10^3^	425
F8	avg	4.41 × 10^3^	6.15 × 10^3^	5.98 × 10^3^	6.51 × 10^3^	6.53 × 10^3^	7.43 × 10^3^	3.79 × 10^3^	2.31 × 10^3^
F8	median	3.35 × 10^3^	5.95 × 10^3^	5.93 × 10^3^	6.66 × 10^3^	7.26 × 10^3^	7.49 × 10^3^	4.31 × 10^3^	2.31 × 10^3^
F9	min	3.00 × 10^3^	3.00 × 10^3^	3.17 × 10^3^	3.09 × 10^3^	2.95 × 10^3^	3.59 × 10^3^	2.90 × 10^3^	2.88 × 10^3^
F9	std	134.00	11.20	98.10	121.00	76.70	90.60	38.40	47.20
F9	avg	3.19 × 10^3^	3.03 × 10^3^	3.32 × 10^3^	3.26 × 10^3^	3.07 × 10^3^	3.69 × 10^3^	2.96 × 10^3^	2.96 × 10^3^
F9	median	3.16 × 10^3^	3.03 × 10^3^	3.33 × 10^3^	3.24 × 10^3^	3.05 × 10^3^	3.67 × 10^3^	2.96 × 10^3^	2.96 × 10^3^
F10	min	2.90 × 10^3^	2.89 × 10^3^	3.73 × 10^3^	2.94 × 10^3^	2.88 × 10^3^	2.90 × 10^3^	2.89 × 10^3^	2.89 × 10^3^
F10	std	71.20	1.77	226.00	956.00	17.80	11.60	15.60	14.50
F10	avg	2.99 × 10^3^	2.89 × 10^3^	4.13 × 10^3^	3.43 × 10^3^	2.89 × 10^3^	2.93 × 10^3^	2.91 × 10^3^	2.91 × 10^3^
F10	median	2.98 × 10^3^	2.89 × 10^3^	4.08 × 10^3^	3.01 × 10^3^	2.89 × 10^3^	2.93 × 10^3^	2.90 × 10^3^	2.90 × 10^3^

**Table 3:** Comparison table of optimization results for welded beam.

Welded Beam	PSO	DE	DBO	BKA	SSA	FOX	GCRA	CEGCRA
worst	1.6716	1.7204	1.6823	1.6708	1.6702	1.7171	1.8342	1.6703
best	2.6702	2.1125	1.9048	3.0260	1.8167	2.3555	2.1740	1.9585
std	0.3115	0.1442	0.0619	0.4267	0.0689	0.1885	0.1026	0.0889
mean	1.9632	1.8886	1.7694	1.8519	1.7224	2.0578	1.9777	1.7074
median	1.8911	1.8537	1.7644	1.6730	1.6743	2.0873	1.9743	1.6773

**Table 4:** Comparison table of optimization results for cantilever beam design.

Cantilever Beam	PSO	DE	DBO	BKA	SSA	FOX	GCRA	CEGCRA
best	1.339971	1.340066	1.340009	1.339969	1.339962	1.339961	1.339961	1.339959
worst	1.340225	1.340399	1.341568	1.340016	1.340006	1.339999	1.340094	1.340004
std	8.61 × 10^−5^	0.000116	0.000488	1.4 × 10^−5^	1.75 × 10^−5^	1.34 × 10^−5^	4.42 × 10^−5^	2.94 × 10^−5^
mean	1.340063	1.340177	1.340496	1.339995	1.339987	1.339979	1.340019	1.340001
median	1.340038	1.340122	1.340342	1.339997	1.339989	1.33998	1.34002	1.340001

## Data Availability

The data that support the findings of this study are available from the corresponding author upon request. There are no restrictions on data availability.
